# A bidirectional Mendelian randomized study of classical blood lipids and venous thrombosis

**DOI:** 10.1038/s41598-023-31067-z

**Published:** 2023-03-08

**Authors:** Liu Lin, Pan Luo, Mingyi Yang, Jiachen Wang, Weikun Hou, Peng Xu

**Affiliations:** Department of Joint Surgery, HongHui Hospital, Xi’an Jiaotong University, Xi’an, 710054 Shaanxi China

**Keywords:** Genetics, Diseases, Risk factors

## Abstract

There is still some controversy about the relationship between lipids and venous thrombosis (VTE). A bidirectional Mendelian randomization (MR) study was conducted to clarify the causal relationship between three classical lipids (low-density lipoprotein (LDL), high-density lipoprotein (HDL) and triglycerides (TGs)) and venous thromboembolism (VTE) (deep venous thrombosis (DVT) and pulmonary embolism (PE)). Three classical lipids and VTE were analysed by bidirectional Mendelian randomization (MR). We used the random effect inverse variance weighted (IVW) model as the main analysis model and the weighted median method, simple mode method, weighted mode method and MR–Egger methods as supplementary methods. The leave-one-out test was used to determine the influence of outliers. The heterogeneity was calculated by using Cochran Q statistics in the MR–Egger and IVW methods. The intercept term in the MR‒Egger regression was used to indicate whether horizontal pleiotropy affected the results of the MR analysis. In addition, MR-PRESSO identified outlier single-nucleotide polymorphisms (SNPs) and obtained a stable result by removing outlier SNPs and then performing MR analysis. When we used three classical lipids (LDL, HDL and TGs) as exposure variables, no causal relationship between them and VTE (DVT and PE) was found. In addition, we did not find significant causal effects of VTE on the three classical lipids in reverse MR analysis. There is no significant causal relationship between three classical lipids (LDL, HDL and TGs) and VTE (DVT and PE) from a genetic point of view.

## Introduction

Venous thromboembolism (VTE) is a complex disease that includes deep venous thrombosis (DVT) and its most dangerous complication, pulmonary embolism (PE)^1^. There are many risk factors for DVT, such as trauma, cancer or gene mutations, that promote blood hypercoagulability^2^. The Virchow triad is generally considered to be the main pathogenesis of VTE, including blood flow disturbance, a hypercoagulable state of blood and a procoagulant state of the blood vessel wall^3^. VTE affects millions of people around the world, and more importantly, severe PE can seriously threaten the lives of patients. VTE is associated with a considerable disease burden, which continues to grow as the global population lives longer^4,5^.

In fact, many factors affect the occurrence and development of VTE. In addition to trauma, cancer and other factors, researchers have found that lipids, such as low-density lipoprotein (LDL), high-density lipoprotein (HDL) and triglycerides (TGs), may also affect VTE. A meta-analysis of a case‒control study and a cohort study found that HDL and TGs were significantly associated with venous thrombosis^6^. Another study found that elevated LDL cholesterol levels were only associated with VTE in men^7^. However, in view of the heterogeneity observed in these studies, the results of the meta-analysis must be carefully interpreted. Other studies have found that LDL, HDL and TGs have no effect on VTE^8^. Therefore, there is still some controversy about the relationship between lipids and VTE.

Mendelian randomization (MR) can use genetic variation as an instrumental variable (IV) of exposure and strengthen the causal inference of the exposure-outcome relationship by reducing confounding factors^9^. MR follows the law of random distribution of genetic variation during conception. Because genotypes precede the progression of the disease and are largely independent of postnatal lifestyle or environmental factors, this technique can minimize confounding factors and avoid deviations caused by reverse causality^10^. MR can avoid some typical biases in observational studies, such as small sample sizes and short follow-up times^11^. Based on the existing genetic database, gene variants that regulate lipids and VTE can be regarded as IVs to further study the causal relationship between lipid levels and VTE risk. And there is no research to prove whether VTE can cause the increase of circulating lipids. Bidirectional Mendelian randomized study refers to the use of two samples of Mendelian randomization method to test whether there is a causal relationship between the two traits. In this study, we used MR analysis to test whether an increase in classical blood lipids can lead to VTE, and then to test whether VTE can lead to an increase in circulating lipids. Therefore, a bidirectional MR study was conducted to clarify the causal relationship between three lipids (LDL, HDL and TGs) and VTE (DVT and PE).

## Methods

We performed bidirectional MR analysis of classical lipids (LDL, HDL and TGs) and VTE (DVT and PE). First, we used classical lipids (LDL, HDL and TGs) as exposure variables to conduct two-sample MR analysis with VTE (DVT and PE). In addition, to further clarify whether VTE affects lipids, we used VTE as an exposure variable to analyse the causal relationship between VTE and classical lipids. The hypothesis of this study is that there is no causal relationship between VTE and classical blood lipids.

**Figure 1:**
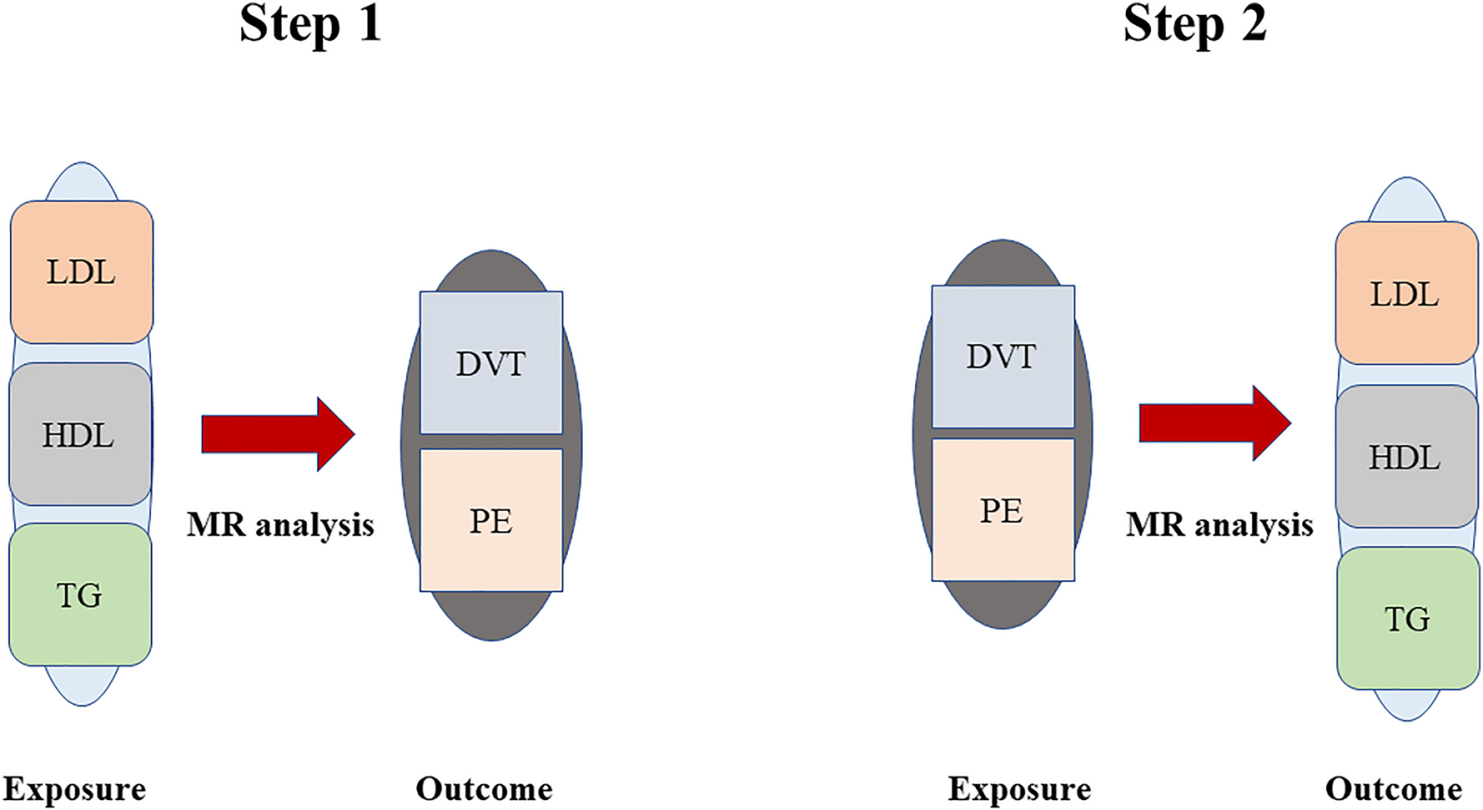
Analysis flow chart. We performed bidirectional MR analysis of classical lipids (LDL, HDL and TG) and VTE (DVT and PE). First, we used classical lipids (LDL, HDL and TG) as exposure variables to conduct two-sample MR analysis with VTE (DVT and PE). In addition, to further clarify whether VTE affects lipids, we used VTE as an exposure variable to analyse the causal relationship between VTE and classical lipids.

The research design is shown in Fig. 1. Since this study is based on existing publications and public databases, no additional ethical approval or consent is needed.

### Data resources

Summary data for DVT were downloaded from the Neale Lab database (http://www.sussex.ac.uk/lifesci/nealelab/) and the MRC IEU OpenGwas repository (https://gwas.mrcieua.ac.uk/). The data of all individual participants were from the UK Biobank study. The DVT data came from a large meta-analysis of a genome-wide association study (GWAS), which included a total of 337,159 subjects of European origin (6767 DVT cases and 330,392 controls). GWAS data for PE were derived from the UK Biobank (41202#I269), including a total of 463,010 subjects of European origin (1846 PE cases and 461,164 controls). The UK Biobank is a large prospective cohort study involving approximately 500,000 people between 37 and 76 years old (99.5 percent between 40 and 69 years old) across the UK^12^. GWAS data for classic lipids (LDL, HDL and TGs) were derived from the MRC IEU OpenGwas repository, including a total of 337,159 subjects of European origin. Sample processing, determination details, genotyping and quality control of these classical lipid GWAS data can be obtained from previously published studies^13^.

### Selection of instrumental variables

When screening IVs, we followed the three basic hypotheses of MR: first, genetic variation should be closely related to exposure; second, variation should not be affected by the confounding factors of the relationship between exposure and outcome; and third, exposure should affect only the outcome (that is, pleiotropy should be eliminated, and exclusion limitation should be satisfied). Therefore, we selected exposure-related SNPs at genome-wide significance (p < 5 × 10^–8^) as IVs. In addition, none of the instrument SNPs were in a state of linkage disequilibrium (LD). We performed the aggregation process (R^2^ < 0.001, large window size = 10,000 kb) to eliminate LD between SNPs. A missing SNP in the LD control group was also deleted. Third, SNPs with a minimum allele frequency (MAF) < 0.01 were removed.

If the SNP for a particular request did not exist in the generated GWAS, a search was conducted for an SNP (agent) in LD with the requested SNP (target). In addition, to test whether there was a weak tool deviation in the IV, we used F statistics^14^ (F = R^2^ (n − k − 1)/k (1 − R^2^), where R^2^ is the exposure variance explained by the selected tool variable (obtained from the MR Steiger directivity test), n is the sample size and k is the total variable). If the F statistic of the IV is much greater than 10, it is very unlikely that the deviation of instrument variables is very small.

### Mendelian randomized analysis

We used the random effect inverse variance weighted (IVW) model (permitting heterogeneity in causal estimates) as the main analysis model and the weighted median method^15^, simple mode method^16,17^, weighted mode method^16,18^ and MR–Egger method^19^ as supplementary methods^20^. The random effect IVW method regresses genetic associations with the outcome on associations with exposure and fixes the intercept at zero. It provides robust causal estimates when there is heterogeneity and in the absence of horizontal pleiotropy^21,22^. Since there is heterogeneity and no horizontal pleiotropy in this analysis, we take the results of random effect IVW as the main results. The weighted median method provides consistent causal estimates when the effective tool has more than 50% of the weight^23,16^. The weighted mode-based causal estimate consistently estimates the true causal effect when the largest group of instruments with consistent MR estimates is valid^18^. The MR‒Egger reversion can recognize and adjust for pleiotropy (p for intercept < 0.05). However, this technique produces estimations of low precision^24^.

### Pleiotropy and sensitivity analysis

We employed MR‒Egger regression to assess the potential pleiotropic effects of IVs. The intercept term in the MR‒Egger regression can be used to indicate whether horizontal pleiotropy affects the results of the MR analysis^24^. MR-PRESSO is a method for the detection and correction of outliers in IVW linear regression. MR-PRESSO analysis attempts to reduce heterogeneity in causal effect estimation by removing SNPs that cause greater than expected heterogeneity. The heterogeneities were quantified by the Cochran Q statistic in the IVW method and MR‒Egger regression, with a P value of 0.05 indicating considerable heterogeneity. Additionally, to identify potentially influential SNPs, we performed a leave-one-out sensitivity analysis.

The beta value and odds ratio (OR) show the kind of relationship between exposure variables and outcome variables. A correlation of P < 0.016 for the beta value (0.05/3 exposures) was considered to be significant, and a correlation between p > 0.016 and < 0.05 was considered to be a suggestive correlation. The threshold of p < 0.05 was used in all sensitivity analyses. Investigations were carried out with the MendelianRandomization, TwoSampleMR and MR-PRESSO packages in R version 4.1.2 (2021-11-01)^25^.

## Results

### Selection of tool variables

The details of all independent SNPs associated with exposure after the SNPs of incompatible alleles were removed are shown in Supplementary File 1. The SNP details of calculating F statistics in all analyses can be found in Supplementary File 1. In our study, the F statistics of IVs related to exposure were all greater than 10, indicating that the possibility of variable deviation of weak tool variables was very small.

### Causal relationship between LDL and DVT

#### Causal relationship between LDL (exposure) and DVT (outcome)

**Figure 2:**
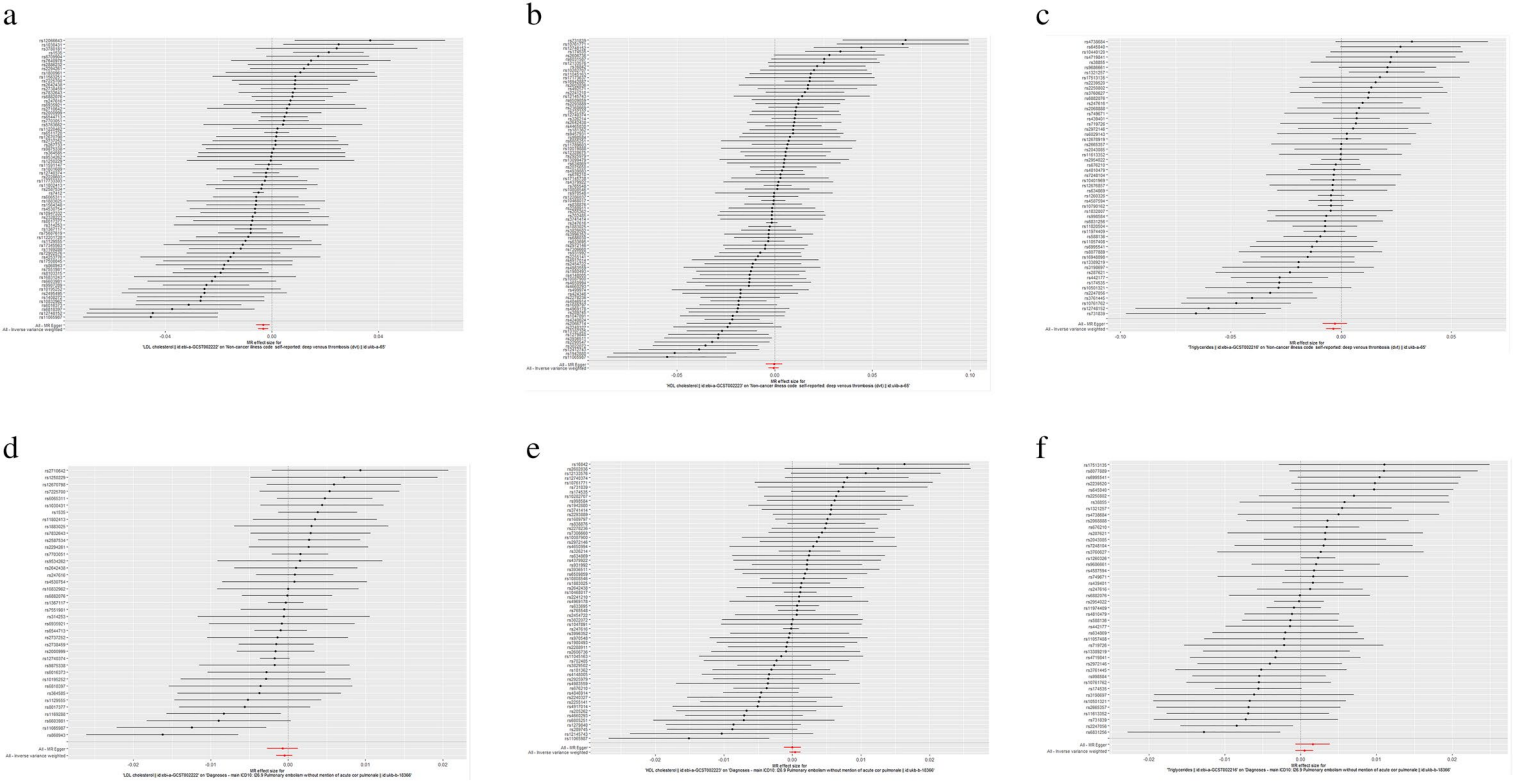
Forest plots of variant specific inverse variance estimates for the causal association between VTE (exposure) and classical lipids (exposure). (**a**) LDL-DVT; (**b**) HDL-DVT; (**c**) TG-DVT; (**d**) LDL-PE; (**e**) HDL-PE, (**f**) TG-PE.

As shown in Table 1, in the MR analysis of LDL and DVT when LDL was used as an exposure variable, according to the results of IVW, there was a causal relationship between an increase in LDL level and a decrease in DVT risk (beta = − 0.003, 95% CI (− 0.005, − 0.001); OR = 0.996, 95% CI (0.994, 0.998); P_beta_ = 9.925e−4) (Fig. 2a). In addition, the P values of the weighted median (beta = − 0.004, 95% CI (− 0.006, − 0.002); P_beta_ = 2.136e−5), MR–Egger (beta = − 0.003, 95% CI (− 0.005, − 0.002); P_beta_ = 0.0143) and weighted mode (beta = − 0.003, 95% CI (− 0.005, − 0.002); P_beta_ = 7.058e−5) methods were all less than 0.05. Only the P value of the simple mode (beta = − 0.001, 95% CI (− 0.007, 0.003); P_beta_ = 0.513) method was greater than 0.05. The heterogeneity analysis found that there was heterogeneity in the analysis (the Q-p values of IVW and MR–Egger were both less than 0.05). The MR–Egger intercept showed that there was no horizontal pleiotropy in the analysis (MR–Egger intercept p value > 0.05) (Table 1). The MR-PRESSO results showed that there were some SNPs that affected the stability of the results, and after removing these SNPs, it was found that LDL had no effect on DVT (Table 2). The scatter plots and funnel plots are shown in Supplementary File 2, Figs. S1 and S7. The leave-one-out test plot showed that there were no SNPs affecting the result (Supplementary File 2, Fig. S13).

**Table 1:** MR estimates from different methods of assessing the causal effect of classical lipids on VT.

Exposure-outcome	No. of SNP	IVW					Weighted median		Weighted mode		Simple mode		MR-Egger					
		Beta (95%CI)	OR (95%CI)	P value	Cochran Q statistics (df)	Q-P value	Beta (95%CI)	P value	Beta (95%CI)	P value	Beta (95%CI)	P value	Beta (95%CI)	P value	Intercept (Se)	P value	Cochran Q statistics (df)	Q-P value
LDL-DVT	70	− 0.003 (− 0.005, − 0.001)	0.996 (0.994, 0.998)	9.925e−4	144.31(69)	2.98e−7	− 0.004 (− 0.006, − 0.002)	2.136e−5	− 0.003 (− 0.005, − 0.002)	7.058e−5	− 0.001 (− 0.007, 0.003)	0.513	− 0.003 (− 0.005, − 7e−4)	0.0143	6.48e−6 (9.86e−5)	0.947	144.30 (68)	2.01e−7
HDL-DVT	86	− 5e−4 (− 0.003, 0.002)	0.999 (0.996, 1.002)	0.669	208.32 (85)	2.39e−12	− 0.001 (− 0.004, 0.001)	0.246	− 8e−4 (− 0.003, 0.001)	0.460	0.002 (− 0.003, 0.008)	0.374	− 3e−4 (− 0.004, 0.003)	0.870	− 1.36e−5 (1e−4)	0.901	208.28 (84)	1.52e−12
TG-DVT	54	− 0.003 (− 0.006, − 1e−4)	0.996 (0.993, 0.999)	0.038	125.71(53)	7.62e−8	− 0.004 (− 0.007, − 8e−4)	0.013	− 0.003 (− 0.006, − 3e−4)	0.034	− 0.003 (− 0.009, 0.002)	0.305	− 0.002 (− 0.008, 0.002)	0.335	− 4.49e−5 (1e−4)	0.740	125.44 (52)	5.22e−8
LDL-PE	39	− 4e−4 (− 0.001, 5e−4)	0.999 (0.998, 1.0005)	0.382	49.60 (38)	0.09	− 5e−4 (− 0.001, 8e−4)	0.462	− 7e−4 (− 0.002, 5e−4)	0.275	− 3e−4 (− 0.002, 0.002)	0.809	− 6e−4 (− 0.002, 0.001)	0.500	1.30e−5 (4.89e−5)	0.791	49.51 (37)	0.08
HDL-PE	61	3.641 (− 4e−4, 0.001)	1.0003 (0.999, 1.001)	0.382	77.41 (60)	0.06	− 1.472 (− 0.001, 9e−4)	0.783	3.100 (− 6e−4, 0.001)	0.520	1.009 (− 0.001, 0.003)	0.445	3.300 (− 0.001, 0.001)	0.995	2.35e−5 (3.13e−5)	0.454	76.67 (59)	0.06
TG-PE	44	4e−4 (− 6.872, 0.001)	1.0004 (0.999, 1.001)	0.419	56.33 (43)	0.08	0.001 (6.561, 0.003)	0.041	0.001 (− 1.056, 0.002)	0.075	0.001 (− 1.848, 0.004)	0.422	0.001 (− 6.662, 0.003)	0.173	− 5.26e−5 (4.66e−5)	0.265	54.67 (42)	0.09

*Se* standard error, *SNP* single nucleotide polymorphism, *MR* Mendelian randomization, *IVW* inverse variance weighting, *VT* venous thromboembolism, *PE* pulmonary embolism, *DVT* deep venous thrombosis, *LDL* Low density lipoprotein, *HDL* High-density lipoprotein, *TG* triglyceride, *CI* confidence interval.

**Table 2:** MR-Presso results (classical lipids—VT).

Exposure-outcome	MR analysis	Casual estimate	SD	T-stat	P-value	Global test-p value	Outliers (snp)
HDL-DVT	Raw	− 5e−4	0.001	− 0.453	0.650	< 0.001	rs10761771 rs11065987 rs12748152 rs174535 rs731839
	Outlier-corrected	− 0.001	0.001	− 1.260	0.210		
LDL-DVT	Raw	− 0.002	0.001	− 2.051	0.461	< 0.001	rs12748152 rs3758348 rs7412
	Outlier-corrected	− 0.002	0.001	− 1.901	0.061		
TG-DVT	Raw	− 0.003	0.001	− 2.067	0.043	< 0.001	rs10761762 rs731839
	Outlier-corrected	− 0.002	0.001	− 1.578	0.120		
TG-PE	Raw	4e−4	5e−4	0.807	0.423	0.08	NA
	Outlier-corrected	NA	NA	NA	NA		
LDL-PE	Raw	− 3e−4	5e−4	− 0.764	0.448	0.128	NA
	Outlier-corrected	NA	NA	NA	NA		
HDL-PE	Raw	3e−4	4e−4	0.916	0.362	0.102	NA
	Outlier-corrected	NA	NA	NA	NA		

*SD* standard deviation, *SNP* single nucleotide polymorphism, *MR-PRESSO* MR-Pleiotropy Residual Sum and Outlier method, *PE* pulmonary embolism, *DVT* deep venous thrombosis, *LDL* low density lipoprotein, *HDL* high-density lipoprotein, *TG* triglyceride.

#### Causal relationship between DVT (exposure) and LDL (outcome)

**Figure 3:**
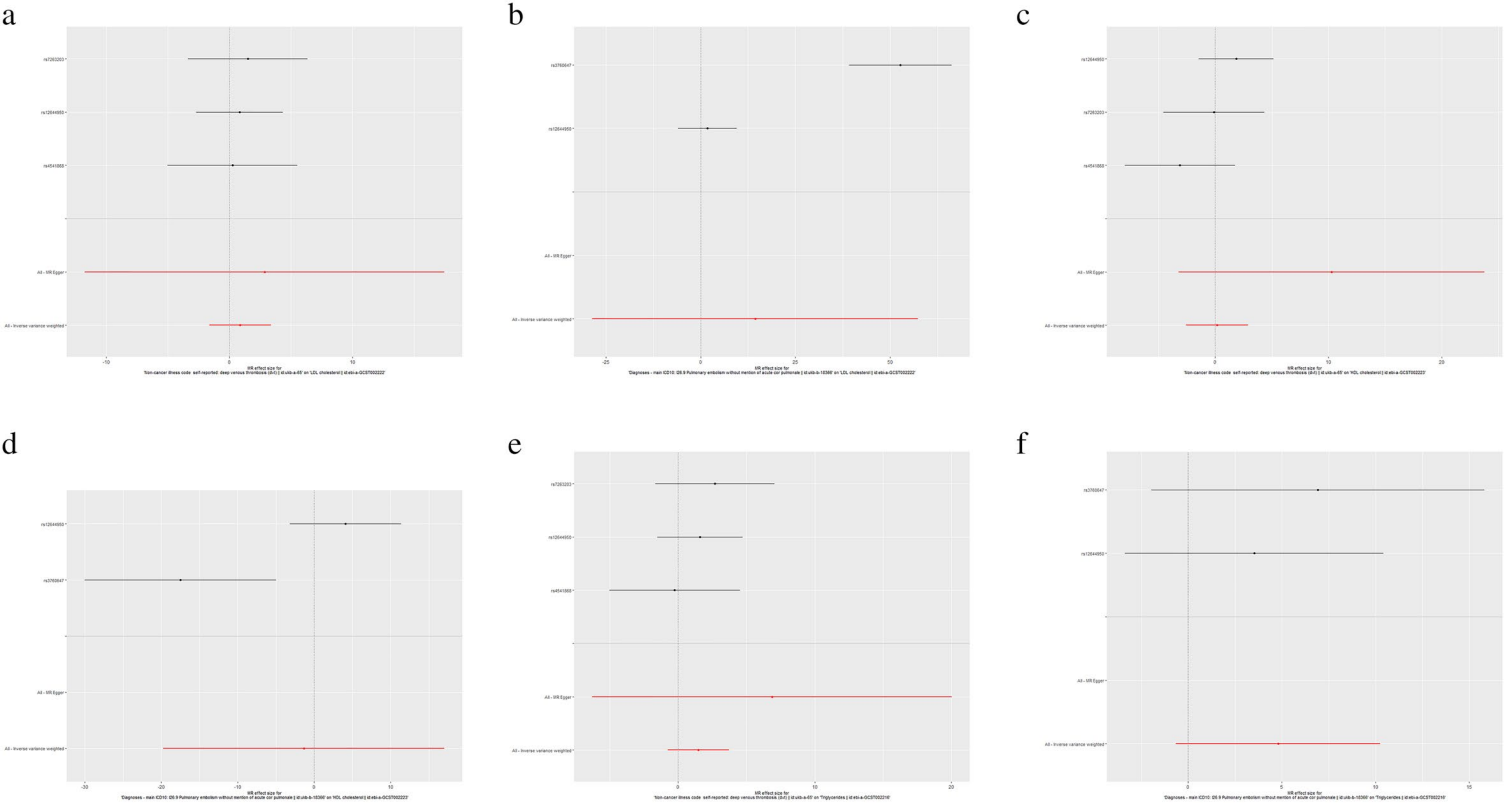
Forest plots of variant specific inverse variance estimates for the causal association between classical lipids (exposure) and VTE (exposure). (**a**) DVT-LDL; (**b**) DVT-HDL; (**c**) DVT-TG; (**d**) PE-LDL; (**e**) PE-HDL; (**f**) PE-TG.

In the MR analysis of LDL and DVT, when DVT was used as an exposure variable, there was no causal relationship between gene-predicted LDL and DVT (the P_beta_ values in all analytical models were greater than 0.05) (Table 3, Fig. 3a). In addition, horizontal pleiotropy analysis and heterogeneity analysis found that the results of this MR analysis were not affected by heterogeneity or horizontal pleiotropy (IVW: Q-P value = 0.943, MR–Egger Q-P value = 0.834, intercept P value = 0.831) (Table 3). MR-PRESSO did not find any SNPs that affected the stability of the results (Table 4). The scatter plot is shown in Supplementary File 2, Fig. S19. The leave-one-out test plot showed that the analysis results were very stable (Supplementary File 2, Fig. S25).

**Table 3:** MR estimates from different methods of assessing the causal effect of VT on classical lipids.

Exposure-outcome	No. of SNP	IVW					Weighted median		Weighted mode		Simple mode		MR-Egger					
		Beta (95%CI)	OR (95%CI)	P value	Cochran Q statistics (df)	Q-P value	Beta (95%CI)	P value	Beta (95%CI)	P value	Beta (95%CI)	P value	Beta (95%CI)	P value	Intercept (Se)	P value	Cochran Q statistics (df)	Q-P value
DVT-LDL	3	0.872 (− 1.631, 3.376)	2.392 (0.195, 29.261)	0.494	0.116 (2)	0.943	0.859 (− 1.864, 3.582)	0.536	0.833 (− 2.404, 4.072)	0.663	0.741 (− 2.432, 3.915)	0.692	2.854 (− 11.740, 17.449)	0.766	0.023	0.831	0.043 (1)	0.834
DVT-HDL	3	0.183 (− 2.546, 2.913)	1.201 (0.078,18.428)	0.895	2.754 (2)	0.252	0.585 (− 2.268, 3.440)	0.687	1.117 (− 1.855, 4.090)	0.538	0.407 (− 3.695, 4.510)	0.863	10.277 (− 3.234, 23.789)	0.376	0.021	0.377	0.545 (1)	0.460
DVT-TG	3	1.482 (− 0.759, 3.724)	4.404 (0.467, 41.449)	0.194	0.807 (2)	0.667	1.645 (− 0.973, 4.264)	0.218	1.766 (− 1.323, 4.856)	0.379	1.913 (− 1.225, 5.052)	0.354	6.872 (− 6.289, 20.035)	0.492	0.021	0.564	0.143 (1)	0.704
PE-LDL	2	14.365 (− 28.58, 57.32)	173 (3.83e−13, 7.83e + 24)	0.512	40.648(1)	1.82e−10	NA	NA	NA	NA	NA	NA	NA	NA	NA	NA	NA	NA
PE-HDL	2	− 3.516 (− 22.19, 15.16)	0.0297 (2.29e−10,384)	0.712	10.8 (1)	0.001	NA	NA	NA	NA	NA	NA	NA	NA	NA	NA	NA	NA
PE-TG	2	4.796 (− 0.656, 10.25)	121.1 (0.518, 28,289)	0.084	0.350 (1)	0.554	NA	NA	NA	NA	NA	NA	NA	NA	NA	NA	NA	NA

*Se* standard error, *SNP* single nucleotide polymorphism, *MR* Mendelian randomization, *IVW* inverse variance weighting, *VT* venous thromboembolism, *PE* pulmonary embolism, *DVT* deep venous thrombosis, *LDL* low density lipoprotein, *HDL* high-density lipoprotein, *TG* triglyceride, *CI* confidence interval.

**Table 4:** MR-Presso results (VT—classical lipids).

Exposure-outcome	MR analysis	Casual estimate	SD	T-stat	P-value	Global test-p value	Outliers (snp)
DVT-LDL	Raw	0.269	0.646	0.417	0.704	0.806	NA
	Outlier-corrected	NA	NA	NA	NA		
DVT-HDL	Raw	− 0.141	1.034	− 1.036	0.899	0.410	
	Outlier-corrected	NA	NA	NA	NA		
DVT-TG	Raw	0.936	0.743	1.258	0.2297	0.680	
	Outlier-corrected	NA	NA	NA	NA		

*SD* standard deviation, *SNP* single nucleotide polymorphism, *MR-PRESSO* MR-Pleiotropy Residual Sum and Outlier method, *DVT* deep venous thrombosis, *LDL* low density lipoprotein, *HDL* high-density lipoprotein, *TG* triglyceride.

### Causal relationship between HDL and DVT

#### Causal relationship between HDL (exposure) and DVT (outcome)

In the MR analysis of HDL and DVT, when HDL was used as an exposure variable, the results of all models showed that there was no causal relationship between the level of HDL and the risk of DVT (P_beta_ value > 0.05 in all models) (Table 1) (Fig. 2b). The heterogeneity test found that there was heterogeneity (the Q-p values of IVW and MR–Egger were both less than 0.05) (Table 1). The MR–Egger intercept showed that there was no horizontal pleiotropy in the analysis (MR–Egger intercept p value > 0.05) (Table 1). The MR-PRESSO results showed that there were some SNPs that affected the stability of the results (MR-PRESSO Global test P value < 0.05), and after removing these SNPs, it was found that HDL had no effect on DVT (Table 2). The leave-one-out test plot did not find problematic SNPs (Supplementary File 2, Fig. S14). The scatter plots and funnel plots are shown in Supplementary File 2, Figs. S2 and S8.

#### Causal relationship between DVT (exposure) and HDL (outcome)

In the MR analysis of HDL and DVT, when DVT was used as an exposure variable, the results of all analytical models showed that DVT did not affect the level of HDL (the P_beta_ values in all models were greater than 0.05) (Table 3, Fig. 3b). The results of the horizontal pleiotropy analysis and heterogeneity analysis showed our MR results were not affected by heterogeneity or horizontal pleiotropic effects (IVW: Q-P value = 0.252, MR–Egger: Q-value = 0.460, intercept P value = 0.377) (Table 3). MR-PRESSO did not find any SNPs that affected the stability of the results (Table 4). The scatter plot is shown in Supplementary File 2, Fig. S20. The analysis chart of the retention method showed no SNPs that affected the results, indicating that the analysis results were stable (Supplementary File 2, Fig. S26).

### Causal relationship between TGs and DVT

#### Causal relationship between TGs (exposure) and DVT (outcome)

As shown in Table 1, when TGs were used as an exposure variable in the MR analysis, according to the results of IVW, there was a genetic causal relationship between TGs and DVT risk reduction (beta = − 0.003, 95% CI (− 0.006, − 1e−4); OR = 0.996, 95% CI (0.993, 0.999); P_beta_ = 0.038) (Fig. 2c). The P values of the weighted median (beta = − 0.004, 95% CI (− 0.007, − 8e−4); P_beta_ = 0.013) and weighted mode (beta = − 0.003, 95% CI (− 0.006, − 3e−4); P_beta_ = 0.034) methods were all consistent with the results of IVW (Table 1). However, the results of the MR–Egger (beta = − 0.002, 95% CI (− 0.008, 0.002); P_beta_ = 0.335) and simple mode (beta = − 0.003, 95% CI (− 0.009, 0.002); P_beta_ = 0.305) methods indicated that the level of TGs did not affect the incidence of DVT (Table 1). The heterogeneity test found that there was heterogeneity (the Q-P values of the IVW and MR–Egger were both less than 0.05) (Table 1). For horizontal pleiotropy analysis, the MR–Egger intercept p value was greater than 0.05 (Table 2); these results indicated that there was no horizontal pleiotropy in this analysis. The results of MR-PRESSO showed that there were some SNP outliers in this analysis. After removing these SNP outliers, it was found that there was no causal relationship between TGs and DVT (Table 2). The results of the leave-one-out test showed that there was no SNP that affected the stability of the results (Supplementary File 2, Fig. S15). The scatter and funnel plots are shown in Supplementary File 2, Figs. S3 and S9.

#### Causal relationship between DVT (exposure) and TGs (outcome)

As shown in Table 3, when DVT was used as an exposure variable in the MR analysis, there was no genetic causal relationship between TGs and DVT risk (the P_beta_ values were greater than 0.05 in all analytical models) (Fig. 3c). The horizontal pleiotropy analysis and heterogeneity analysis indicated that our MR results were not affected by heterogeneity or horizontal pleiotropy (Tables 3, 4). The scatter plot is shown in Supplementary File 2, Fig. S21. The leave-one-out test plot showed that the analysis results were very stable (Supplementary File 2, Fig. S27).

### Causal relationship between LDL and PE

#### Causal relationship between LDL (exposure) and PE (outcome)

In the MR analysis of LDL and PE, when LDL was used as an exposure variable, there was no genetic causal relationship between LDL and PE risk (P_beta_ > 0.05 in all analytical models) (Table 1, Fig. 2d). The p value of the Q statistic was less than 0.05, indicating heterogeneity (Table 1). However, there was no horizontal pleiotropy in this analysis (the MR–Egger intercept p value was greater than 0.05) (Table 1). There was no SNP affecting the stability of the results according to the leave-one-out test (Supplementary File 2, Fig. S16). The scatter plots and funnel plots are shown in Supplementary File 2, Figs. S4 and S10.

#### Causal relationship between PE (exposure) and LDL (outcome)

In the MR analysis of LDL and PE, when PE was used as an exposure variable, because two related IVs were included, only IVW model analysis could be carried out (Table 3). According to the IVW analysis, there was no genetic causal relationship between PE and LDL levels (beta = 14.365, 95% CI (− 28.58, 57.32); P_beta_ = 0.512) (Table 3, Fig. 3d). Heterogeneity analysis showed that there may be some heterogeneity in this analysis (Q-P value of the IVW = 1.82e−10). Since there were only 2 IVs included in this analysis, it was impossible to perform the sensitivity and leave-one-out tests. The scatter plot is shown in Supplementary File 2, Fig. S22.

### Causal relationship between HDL and PE

#### Causal relationship between HDL (exposure) and PE (outcome)

In the MR analysis of HDL and PE, when HDL was used as an exposure variable, there was no genetic causal relationship between LDL and PE risk (P_beta_ > 0.05 in all analytical models) (Table 1, Fig. 2e). The heterogeneity analysis in this study showed that the analysis had heterogeneity (the Q-P values of IVW and MR–Egger were both less than 0.05) (Table 1). However, horizontal pleiotropy analysis showed that there was no horizontal pleiotropy (MR–Egger intercept P value > 0.05) (Table 1). The leave-one-out test showed that no SNP affected the stability of the results (Supplementary File 2, Fig. S17). The scatter plots and funnel plots are shown in Supplementary File 2, Figs. S5 and S11.

#### Causal relationship between PE (exposure) and HDL (outcome)

In the MR analysis of HDL and PE, when PE was taken as the exposure variable, because two related IVs were included, only the IVW model analysis could be carried out. According to the IVW analysis, there was no causal relationship between HDL and PE risk (beta = − 3.516, 95% CI (− 22.19, 15.16); P_beta_ = 0.712) (Table 3, Fig. 3e). The results of heterogeneity analysis showed that there may be some heterogeneity in this analysis (Q-P value of IVW = 0.001) (Table 3). In addition, because there were few IVs included in this analysis, it was impossible to carry out sensitivity analysis and leave-one-out tests. The scatter plot is shown in Supplementary File 2, Fig. S23.

### Causal relationship between TGs and PE

#### Causal relationship between TGs (exposure) and PE (outcome)

In the MR analysis of TGs and PE, when TGs were taken as an exposure variable, there was no genetic causal relationship between TGs and PE risk (P_beta_ > 0.05 in all analytical models) (Table 1, Fig. 2f). Heterogeneity analysis showed that there was heterogeneity (the Q-P values of IVW and MR–Egger were both less than 0.05) (Table 1). However, horizontal pleiotropy analysis showed that there was no horizontal pleiotropy (the P values of the MR–Egger intercept was greater than 0.05) (Table 1). The results of the leave-one-out test show that the results of the analysis are robust (Supplementary File 2, Fig. S18). The scatter plot and funnel plot are shown in Supplementary File 2, Figs. S6 and S12.

#### Causal relationship between PE (exposure) and TGs (outcome)

In the MR analysis of TGs and PE, when PE was used as an exposure variable, because two related IVs were included, only IVW analysis could be carried out (Table 3). According to the IVW analysis, PE did not affect the level of TGs (beta = 4.796, 95% CI (− 0.656, 10.25); P_beta_ = 0.084) (Table 3, Fig. 3f). The results of the heterogeneity analysis showed that there was no heterogeneity (Q-P value of IVW = 0.554). In addition, because there were few IVs included in this analysis, it was impossible to carry out sensitivity analysis and leave-one-out tests. The scatter plot is shown in Supplementary File 2, Fig. S24.

## Discussion

In this study, we used bidirectional MR studies to analyse the causal relationship between VTE (DVT and PE) and classical lipids (LDL, HDL and TGs). Through our MR analysis, we did not find a causal relationship between VTE and classical lipids. Even though the IVW model of some analyses (such as LDL-DVT and TG-DVT) had a P value less than 0.05, after MR-PRESSO removed the outliers, there was no causal relationship between them.

We found that there is no causal relationship between VTE and blood lipids from the point of view of genetics, which provides evidence for clinical research. Of course, the results may vary dependent on population, sample size, genotyping, alternative genetic methods etc. Additionally, there are further issues such as confounding, instrument strength, population stratification that may bias results. At present, there is still some controversy in clinical research on the causal relationship between LDL and VTE. For example, Petter and others have found that there is no correlation between LDL and VTE^26^. However, Dai et al. believed that a higher LDL value is significantly related to an increased risk of DVT in female patients after TKA^27^. Our results showed that the level of classical lipids does not affect the incidence of DVT. These results are consistent with some previous clinical results, such as the results of Morelli et al.'s MEGA study showing that classical lipids (LDL, HDL and TGs) were not associated with the risk of venous thrombosis^8^. In addition, Schouwenburg et al. also found that the level of blood lipids did not affect the risk of VTE^28^. In patients with recurrent VTE, Morelli et al. did not find a causal relationship between lipids and VTE^29^. Although observational control studies and two meta-analyses have shown that hypolipidaemic drugs such as statins can significantly reduce the risk of VTE through mechanisms related to multiple drug effects, it is likely to be a process independent of cholesterol reduction^30^. For example, statins can induce the expression of Krupp-like factor 2, which in turn promotes the expression of thrombomodulin on endothelial cells, thereby enhancing the activity of the protein C anticoagulation pathway^31^. In addition, statins can reduce the level of inflammatory markers^32^ and reduce tissue factor expression and thrombin production^33^.

A previous meta-analysis showed that patients with venous thrombosis had higher average levels of TGs and lower average levels of HDL^6^. However, most of the studies included in this meta-analysis were small case‒control studies that could not control for confounding factors^6^. In the observational study, there are several possible reasons to explain the association between lipids and VTE. First, there were some confounding factors in the patients included, such as obesity. Elevated blood lipids tend to occur in obese patients, especially those with abdominal obesity, which is associated with increased thrombin formation and decreased fibrinolysis^34^. In addition, obesity is associated with inactivity, which is another risk factor for thrombosis^35,36^. Furthermore, blood lipids usually increase with blood sugar, while diabetes is usually associated with increased levels of procoagulant factors and endogenous fibrinolysis inhibition^37,38^. Although some studies have shown that elevated LDL levels accelerate the activation of prothrombin, factor X and factor VII, HDL enhances the protein C anticoagulant pathway and reduces thrombin production^39^. However, this study did not show an association between classical lipids and the risk of VTE. This suggests that the prethrombotic effects of dyslipidaemia may be too mild to truly affect the risk of VTE or that these effects can be offset by other mechanisms. Of course, this needs to be verified by further experiments.

The current bidirectional MR analysis has several advantages. First, this study is the first to infer a causal relationship between classical lipids (LDL, HDL and TGs) and VTE (DVT and PE) from a genetic perspective. Moreover, bidirectional analysis ensured the inference of bidirectional causality between VTE and lipids. However, this study has some limitations. First, the people in the MR analysis were of European descent, so this study may not be applicable to other races. Second, there may be overlapping participants in the exposure and outcome studies, but it is difficult to estimate the extent of sample overlap. In addition, although many cases of VTE were found in the current GWAS analysis, they could not be stratified or adjusted for in the analysis. Of course, larger sample sizes of clinical studies and experiments are needed to confirm our conclusions. We could confirm that an exposure-associated SNP is thus far only reported to be associated with that particular exposure, but we cannot guarantee that the same SNP is not associated with other traits (confounders); the association might remain to be identified, or the SNP might be associated with an underlying risk factor that is unrecognized. Although researchers have proposed many solutions that meet the assumptions of the MR model, these strategies can only detect a violation of the hypothesis but can never confirm whether it is true. Therefore, these shortcomings may lead to biased estimates.

## Conclusion

Through bidirectional MR studies, we found that there was no genetic causal relationship between VTE (DVT and PE) and classical lipids (LDL, HDL and TGs), which laid a foundation for future studies of VTE and classical lipids.

## Supplementary Information

Supplementary Information 1.

Supplementary Information 2.

## Data Availability

Data used in the present study are all publicly available. Corresponding author will provide the data upon reasonable request.

## Abbreviations

VTE: Venous thrombosis
DVT: Deep venous thrombosis
PE: Pulmonary embolism
LDL: Low-density lipoprotein
HDL: High-density lipoprotein
TGs: Triglycerides
SNPs: Single-nucleotide polymorphisms
GWAS: Genome-wide association study
IVW: Inverse-variance weighted
MR: Mendelian randomization
IV: Instrumental variable
CIs: Confidence intervals
MR-PRESSO: MR-Pleiotropy Residual Sum and Outlier method
