# Supplementary material for: A bidirectional Mendelian randomized study of classical blood lipids and venous thrombosis
Source: Sci Rep. 2023 Mar 8;13:3904. doi: 10.1038/s41598-023-31067-z (PMC9995644; doi:10.1038/s41598-023-31067-z)
Supplement: Supplementary file 2 — Supplementary Information 2. [file 41598_2023_31067_MOESM2_ESM.docx]

**Supplemental Figure S1: Scatterplot of the causal relationships between LDL and DVT**


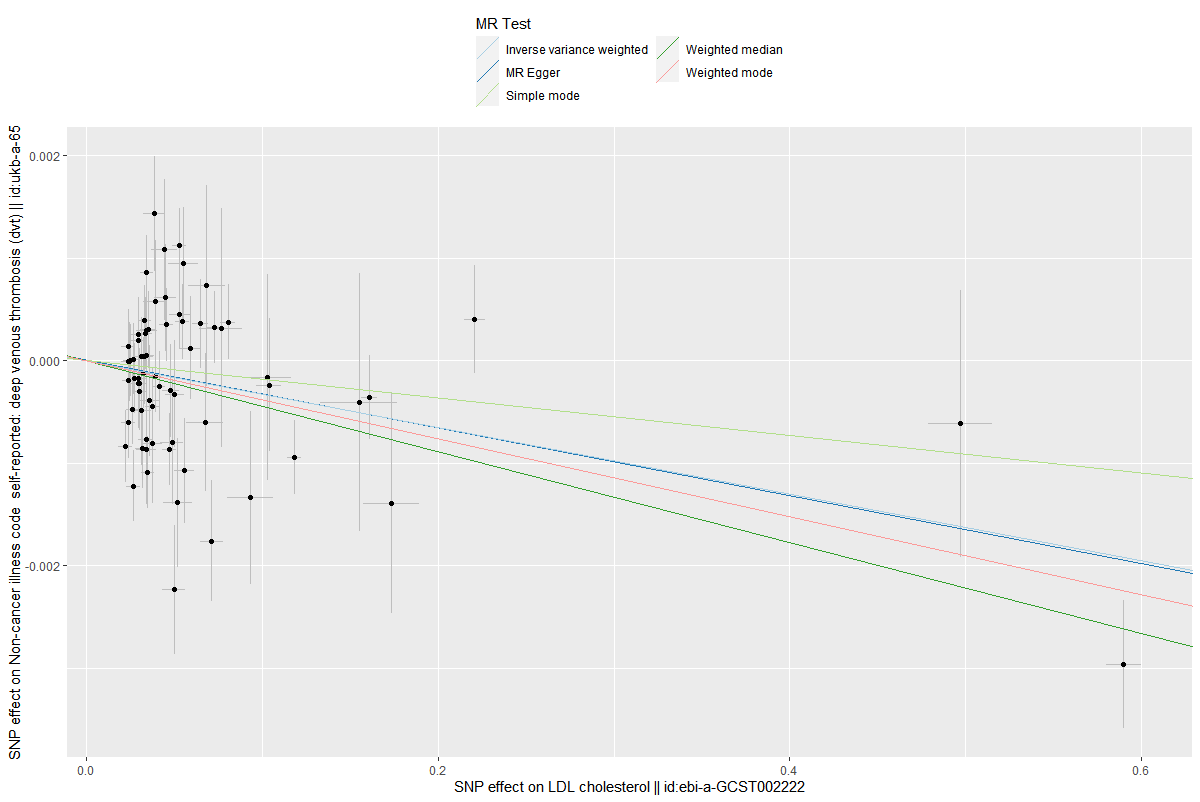


**Supplemental Figure S2: Scatterplot of the causal relationships between HDL and DVT**

**
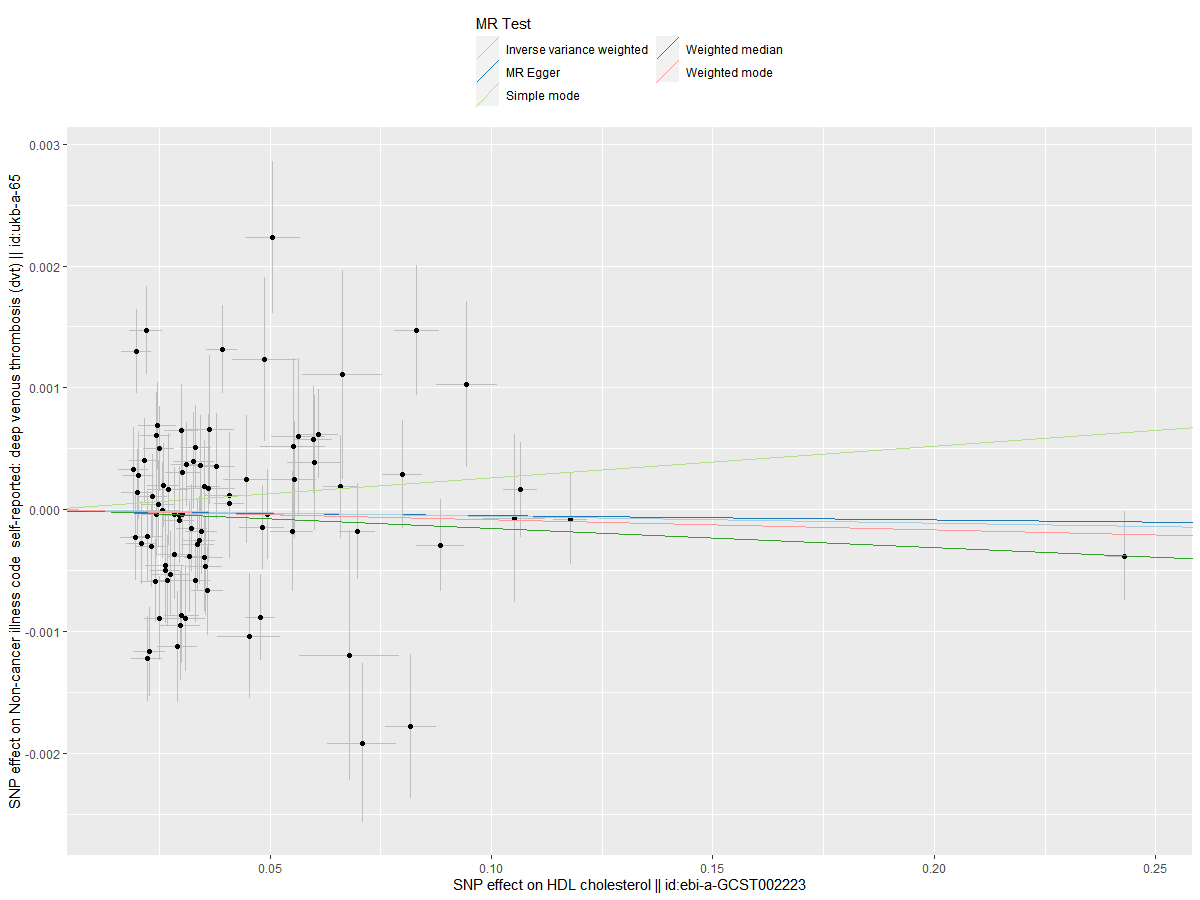
**

**Supplemental Figure S3: Scatterplot of the causal relationships between TG and**

**DVT**

**
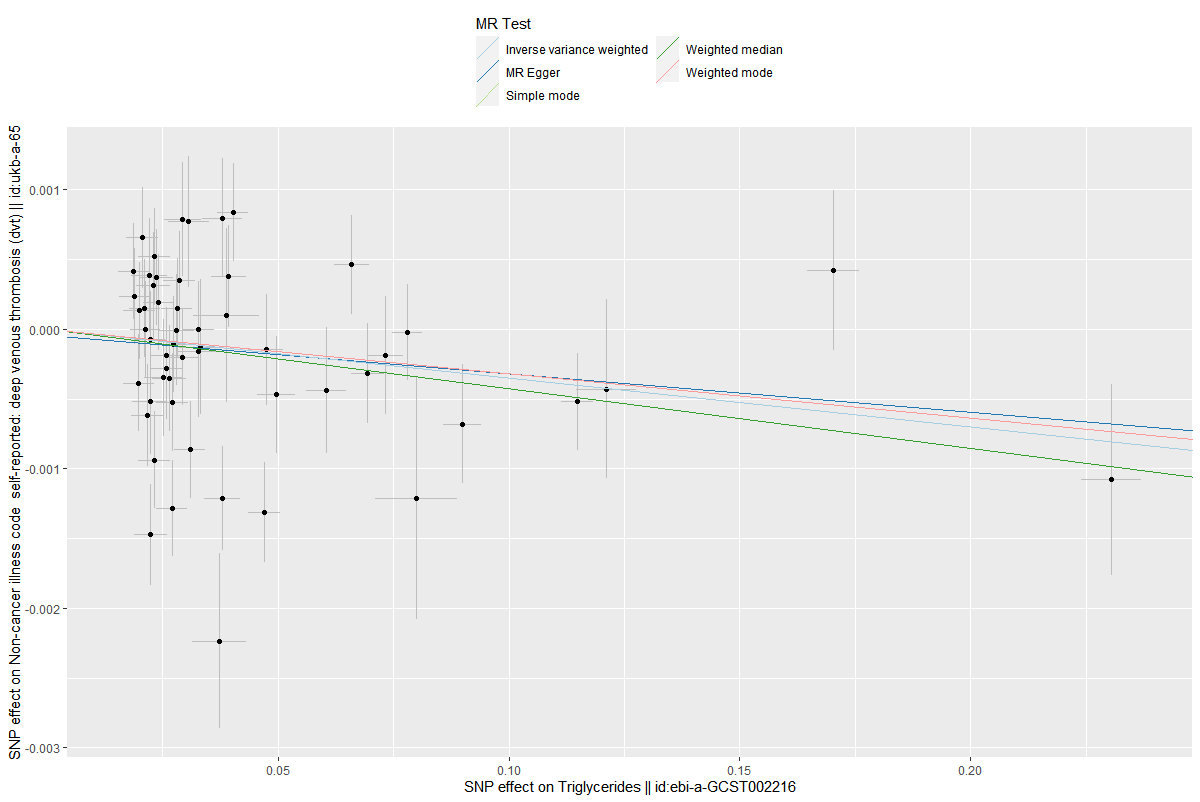
**

**Supplemental Figure S4: Scatterplot of the causal relationships between LDL and PE**

**
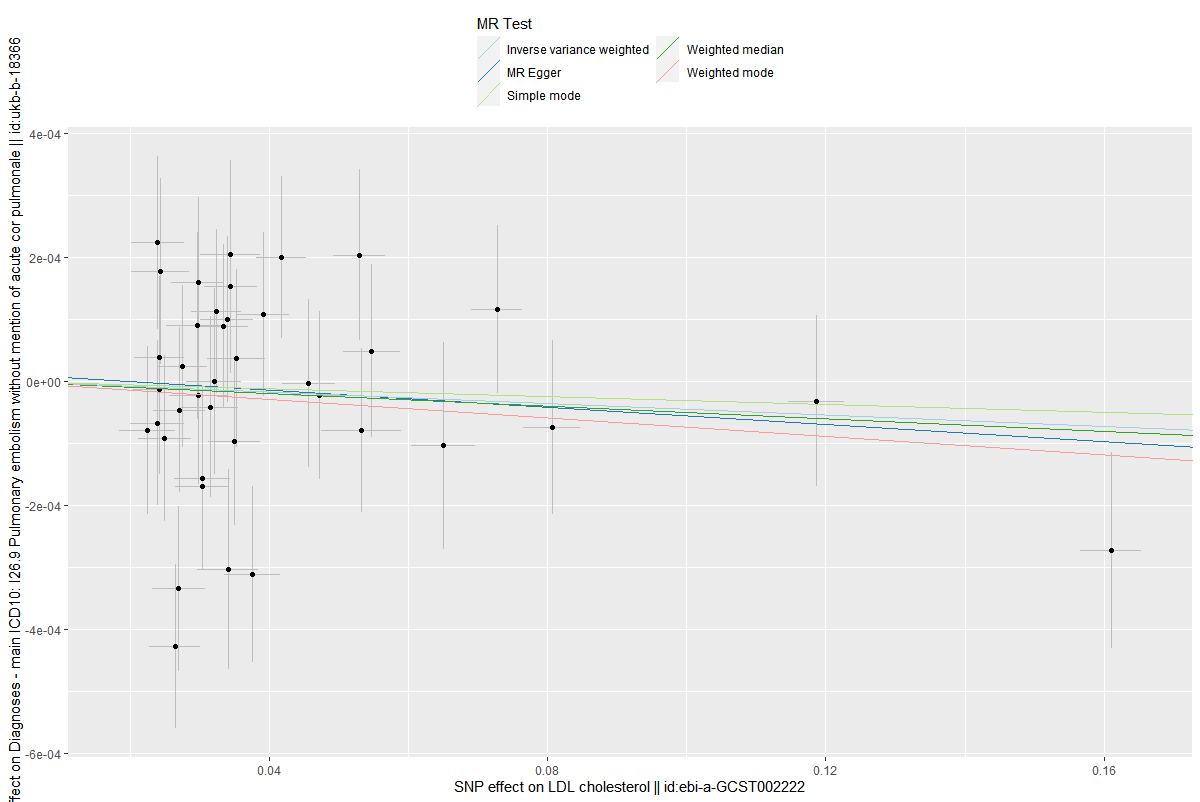
**

**Supplemental Figure S5: Scatterplot of the causal relationships between HDL and PE**

**
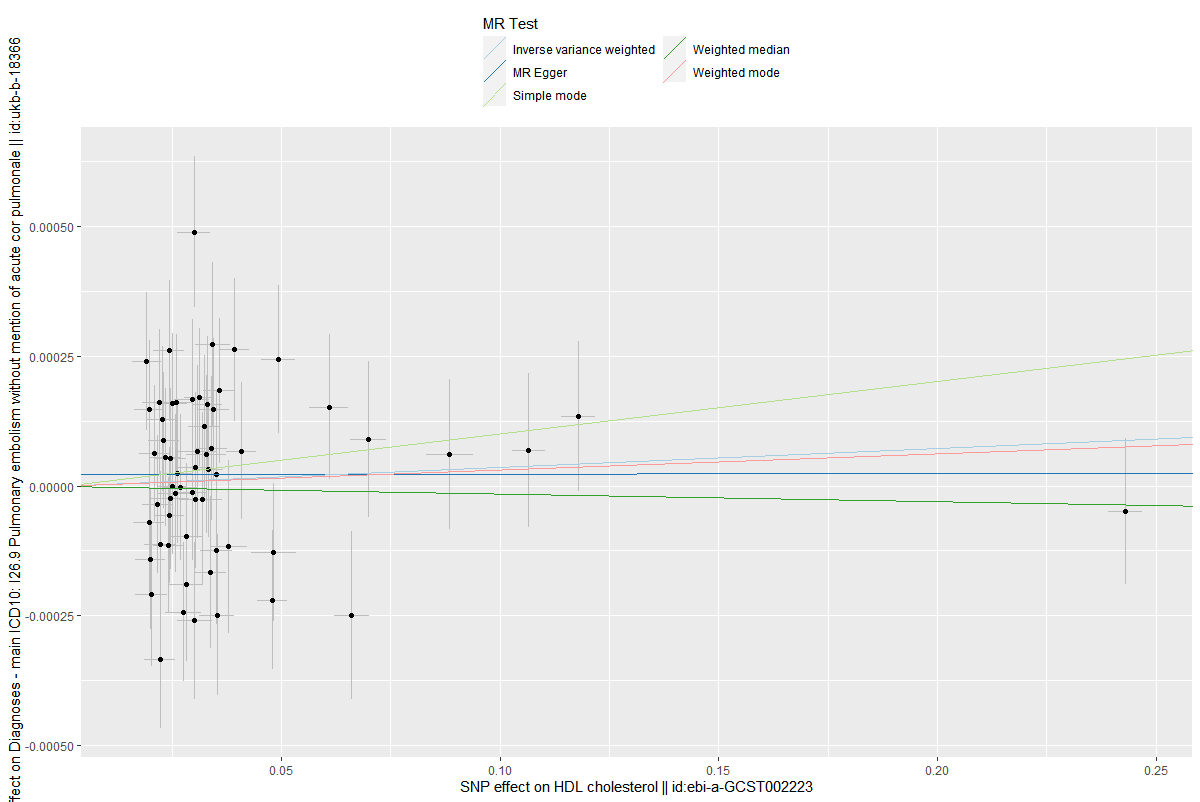
**

**Supplemental Figure S6: Scatterplot of the causal relationships between TG and PE**

**
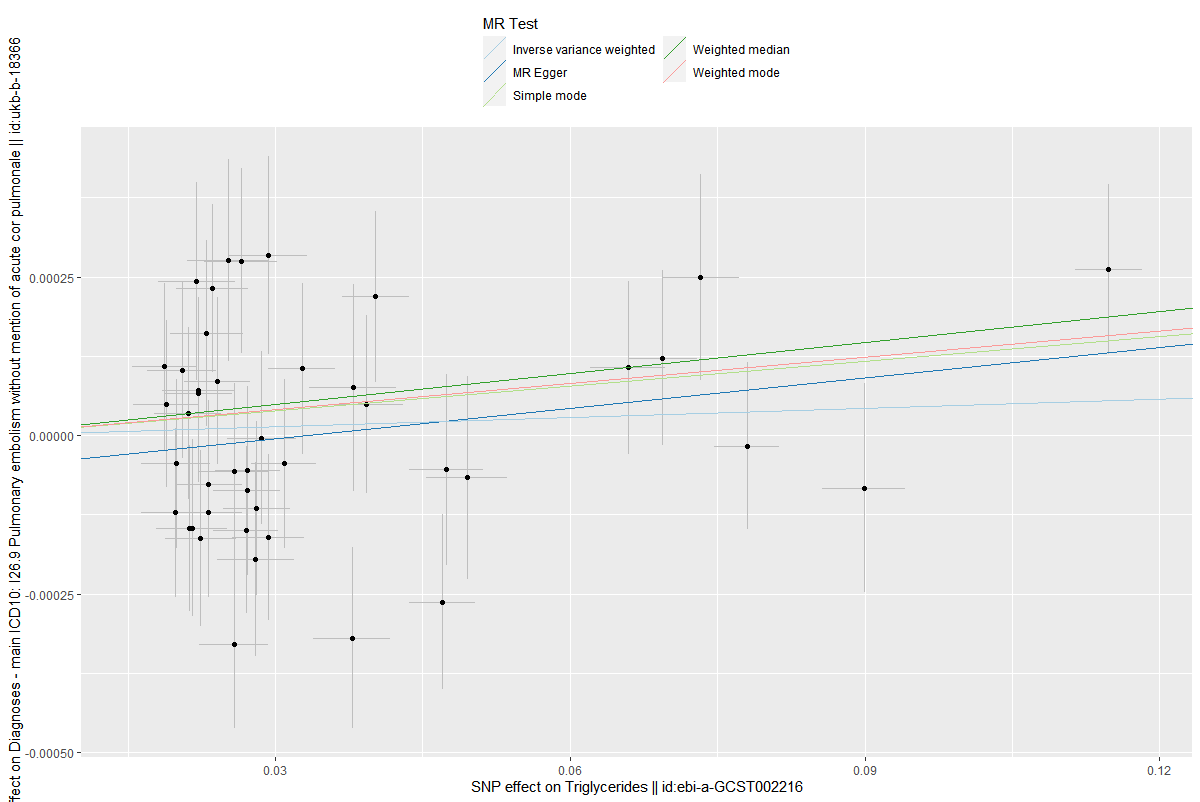
**

**Supplemental Figure S7: Funnel plot of the causal association between LDL and DVT**

**
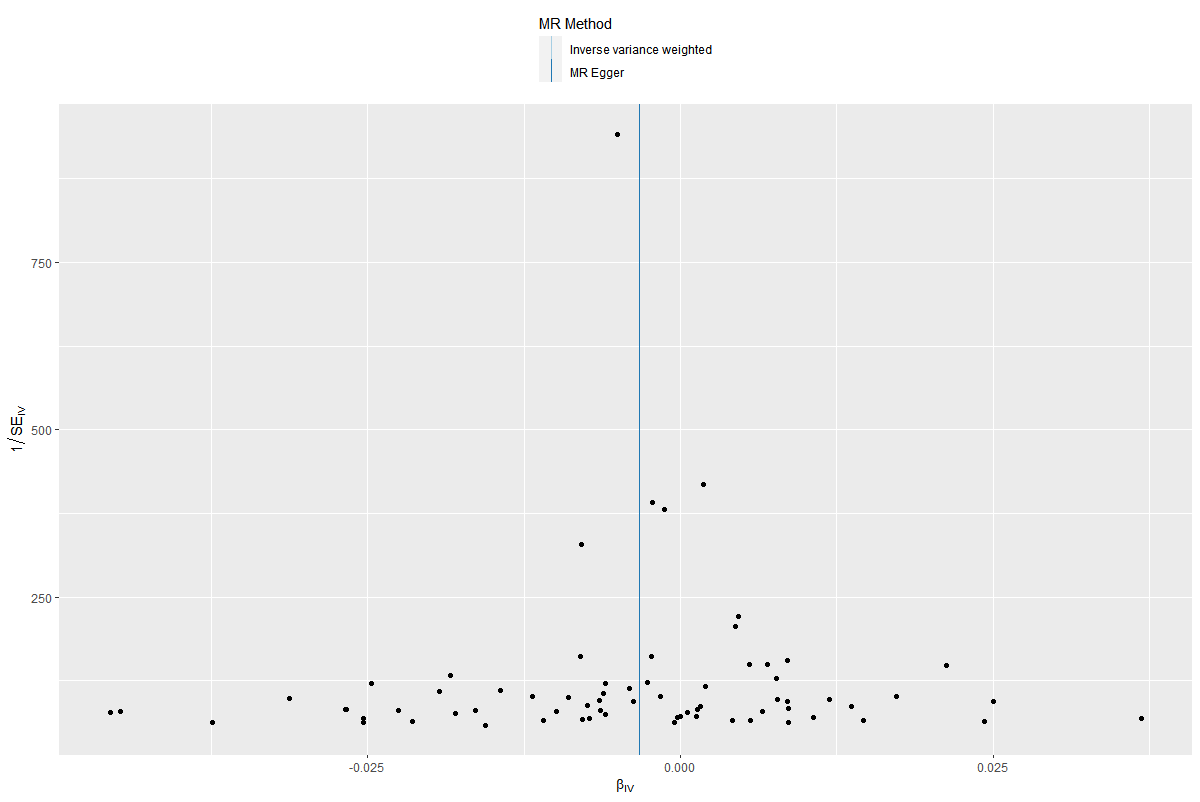
**

**Supplemental Figure S8: Funnel plot of the causal association between HDL**

**and DVT**

**
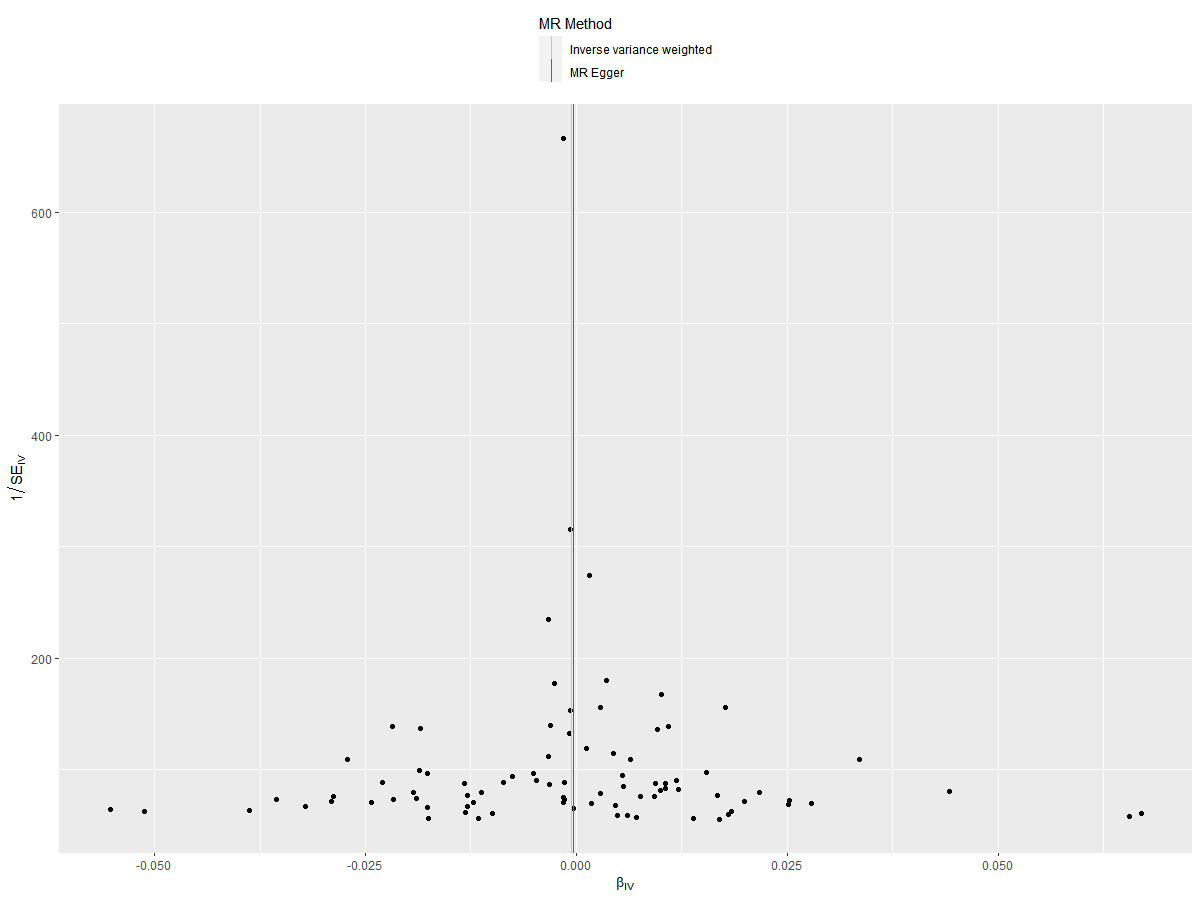
**

**Supplemental Figure S9: Funnel plot of the causal association between TG**

**and DVT**

**
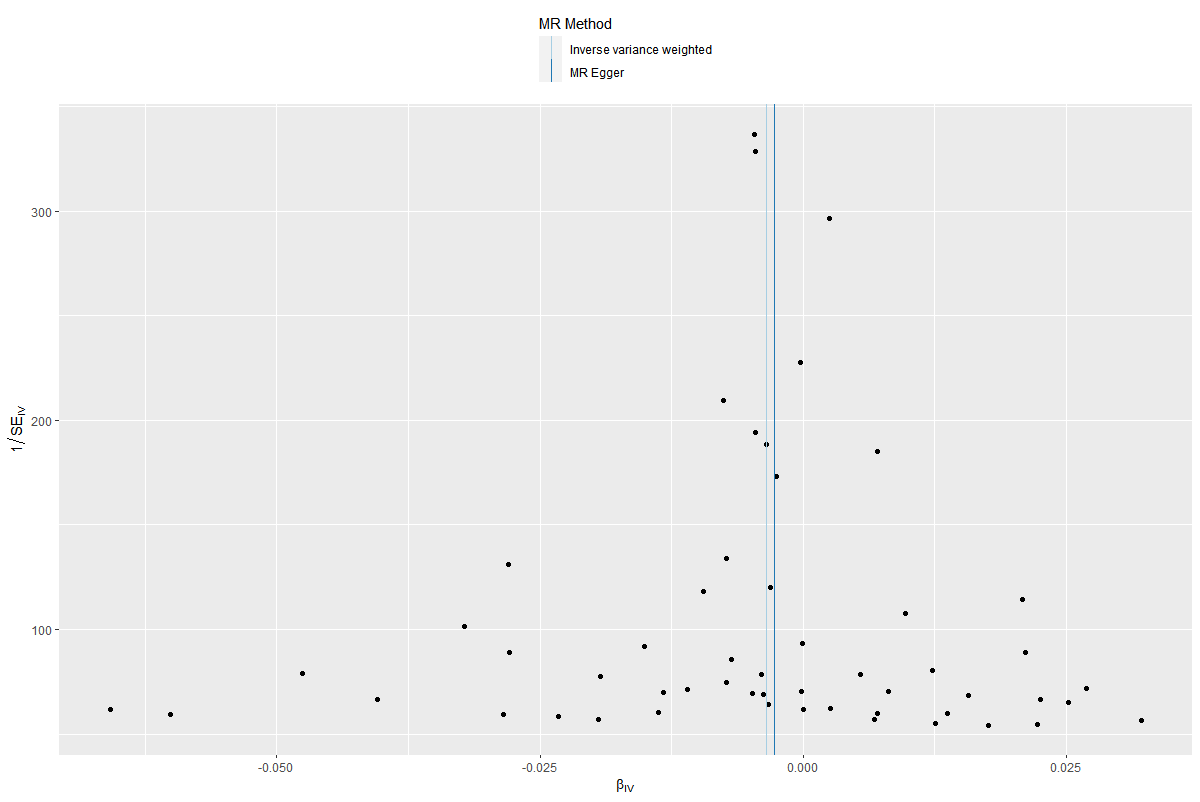
**

**Supplemental Figure S10: Funnel plot of the causal association between LDL and PE**

**
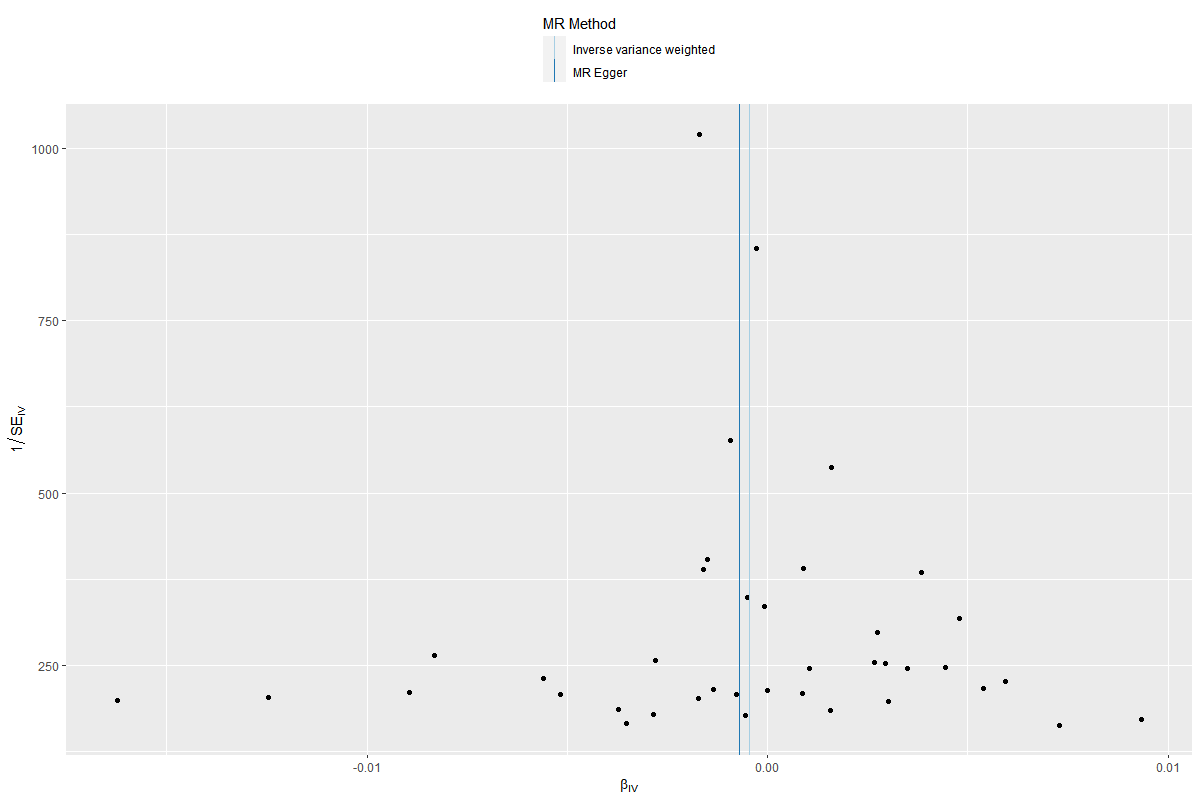
**

**Supplemental Figure S11: Funnel plot of the causal association between HDL and PE**

**
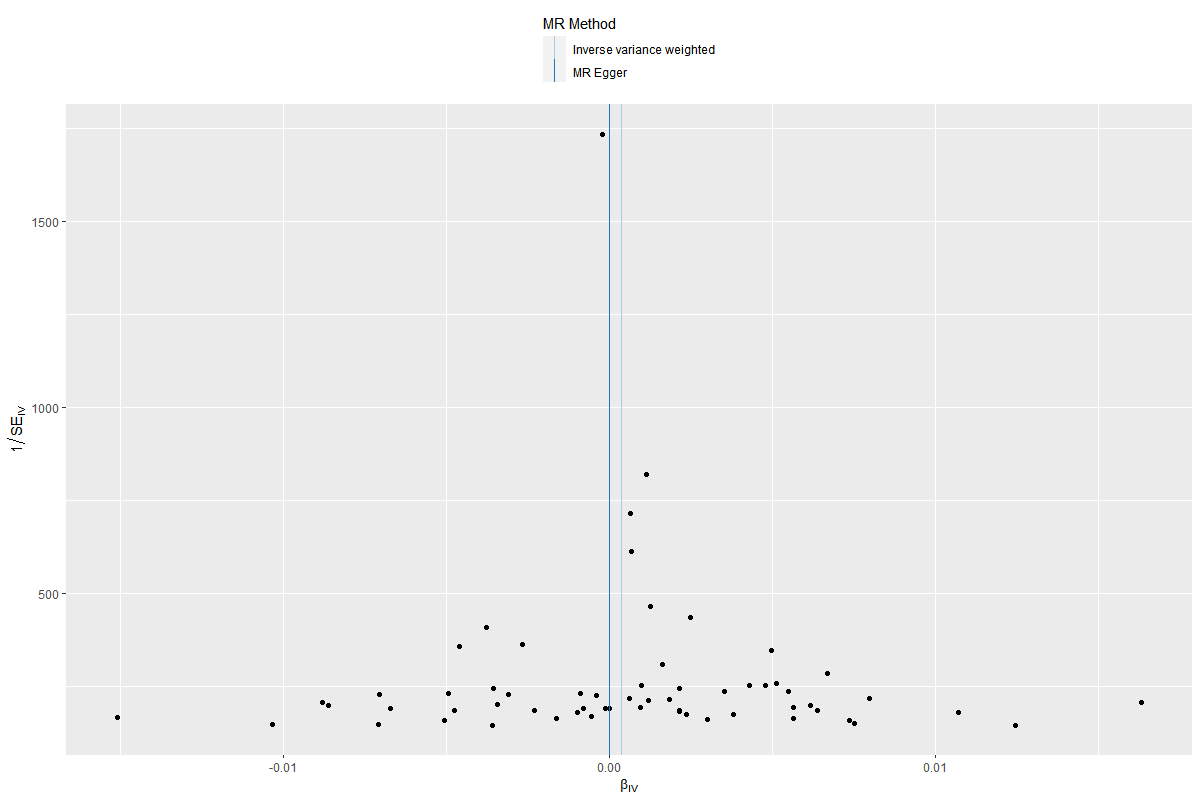
**

**Supplemental Figure S12: Funnel plot of the causal association between TG**

**and PE**

**
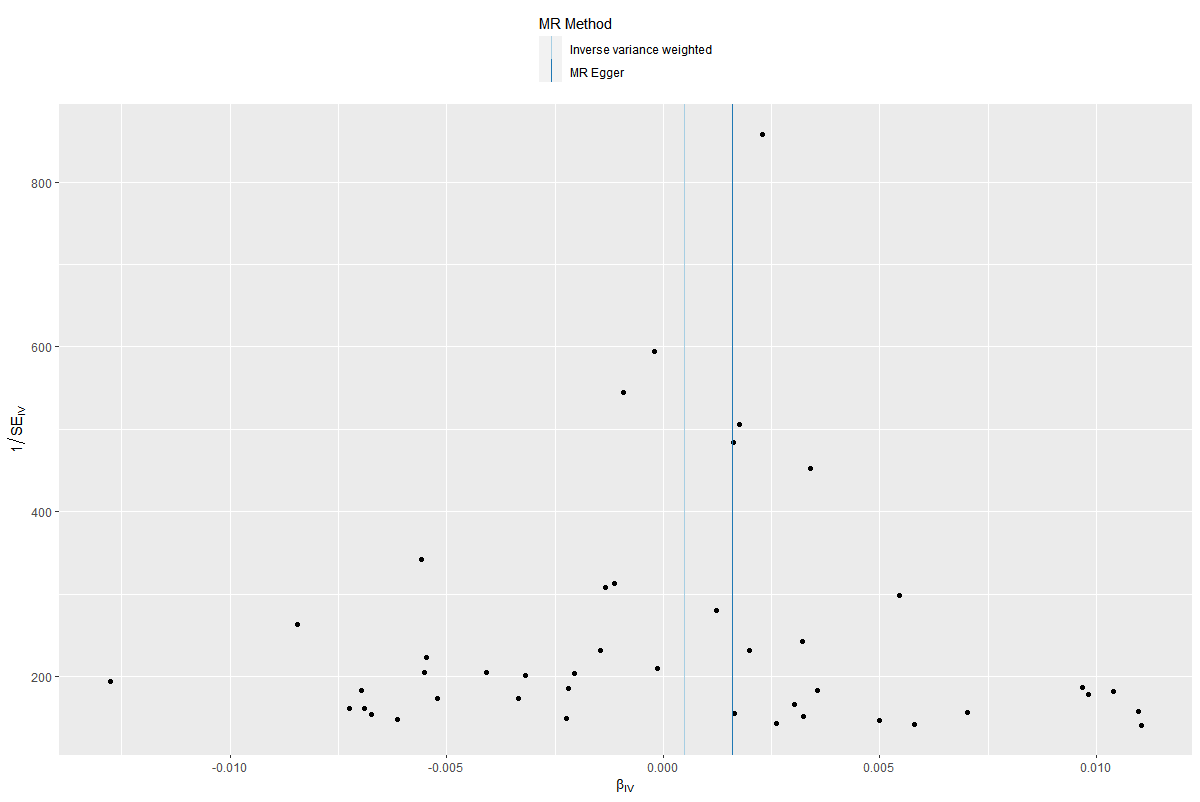
**

**Supplemental Figure S13: Leave-one-out test plot of the causal association between LDL and DVT**

**
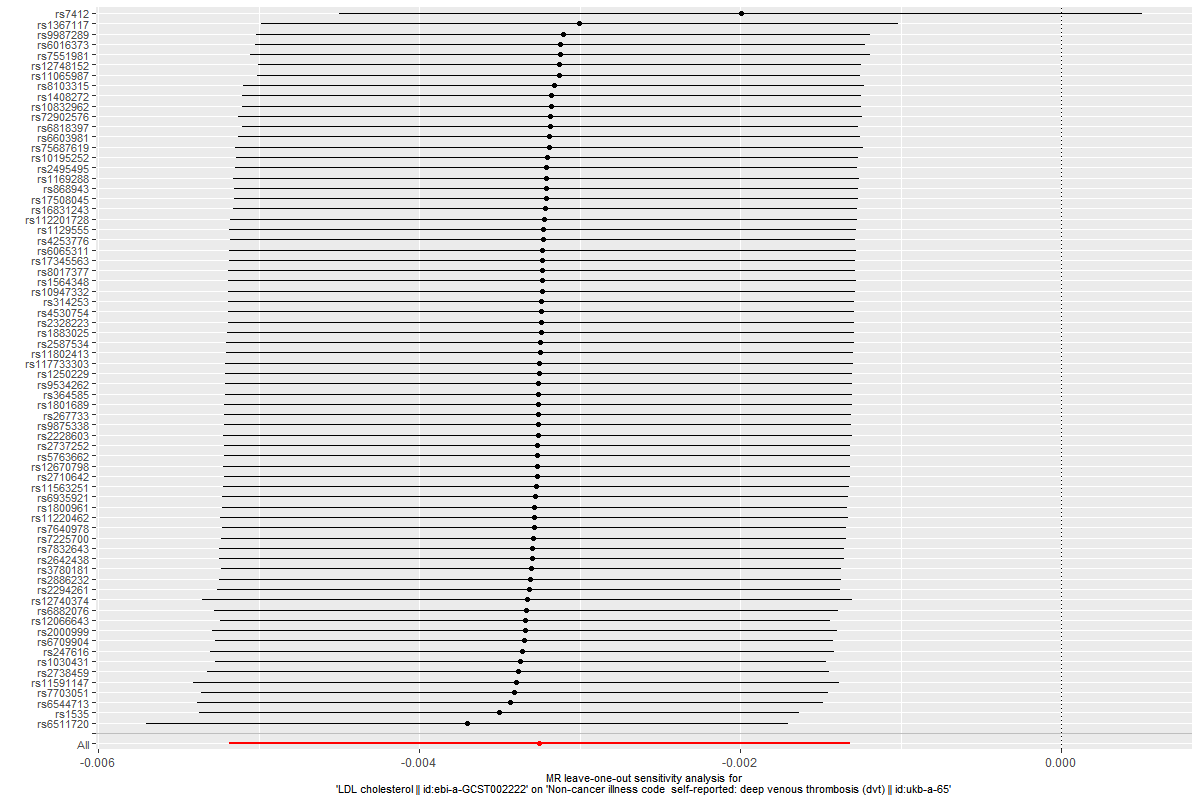
**

**Supplemental Figure S14: Leave-one-out test plot of the causal association between HDL and DVT**

**
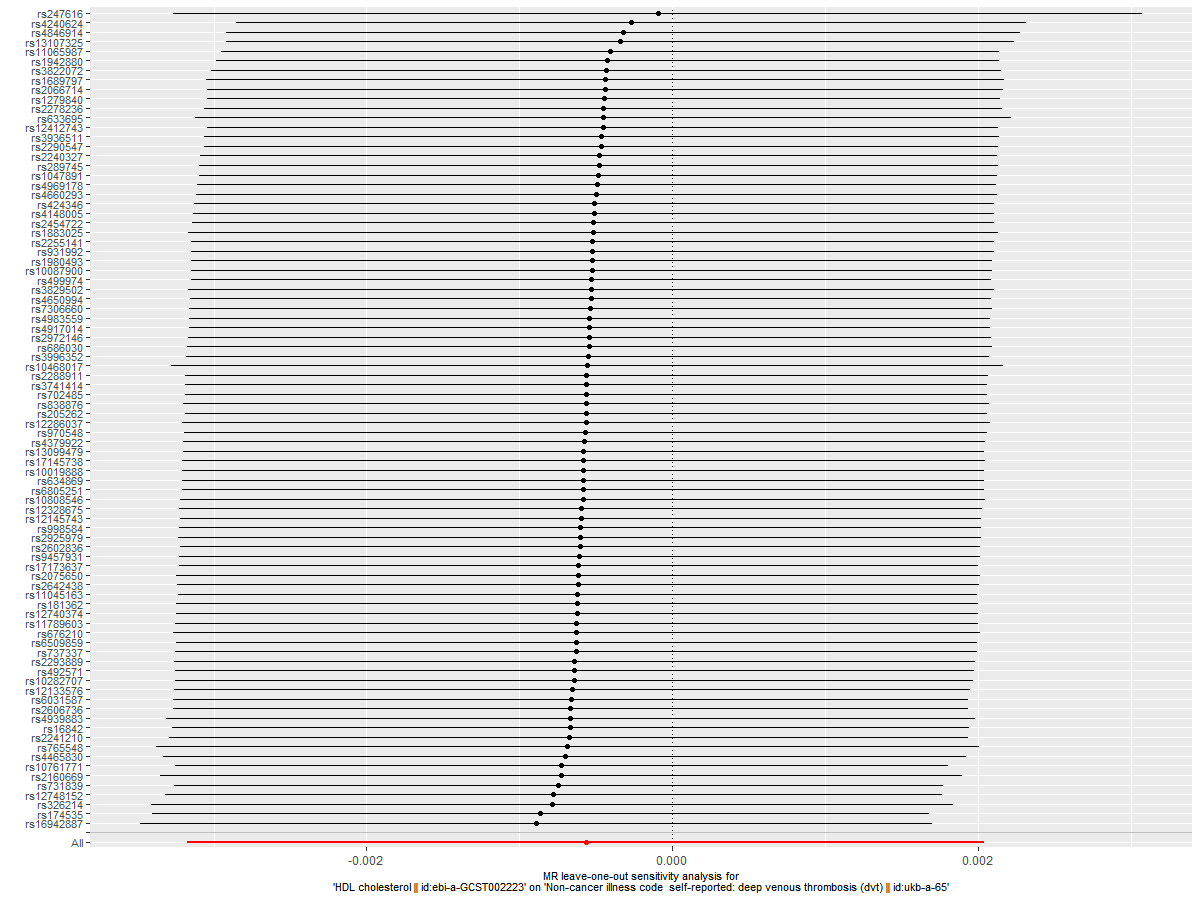
**

**Supplemental Figure S15: Leave-one-out test plot of the causal association between TG and DVT**

**
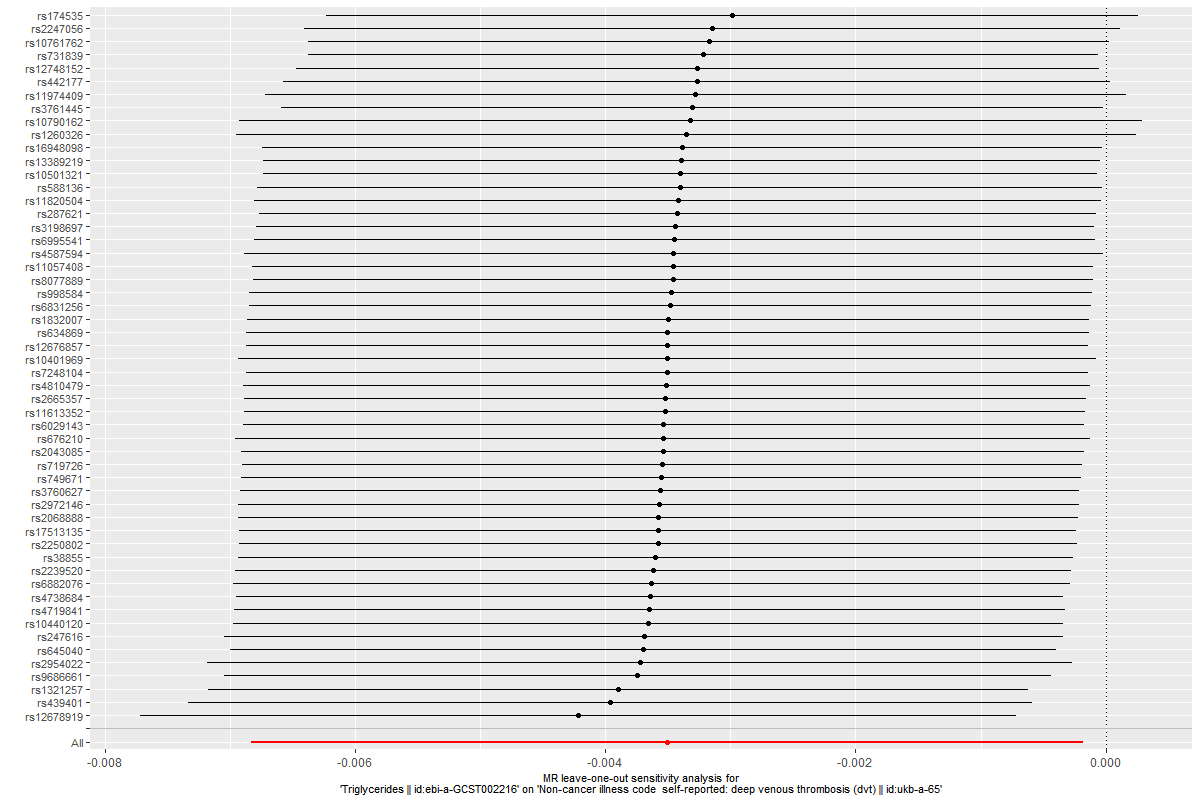
**

**Supplemental Figure S16: Leave-one-out test plot of the causal association between LDL and PE**

**
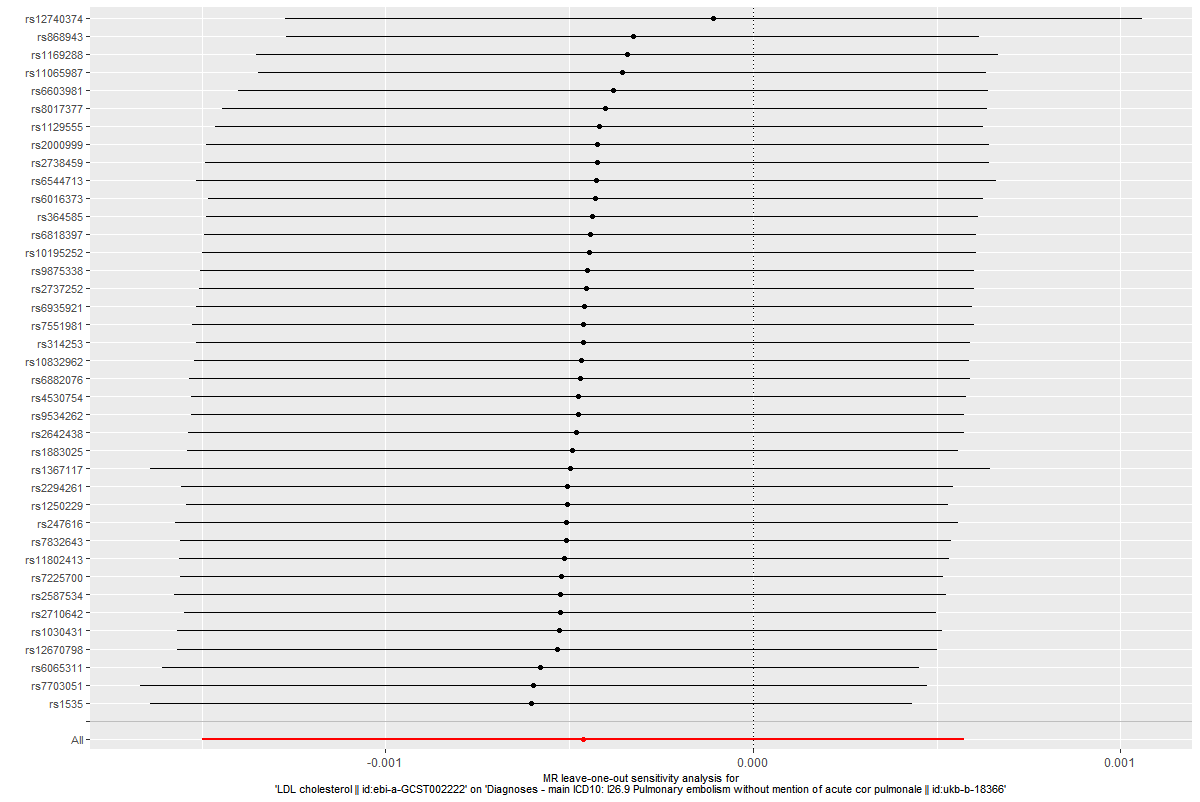
**

**Supplemental Figure S17: Leave-one-out test plot of the causal association between HDL and PE**


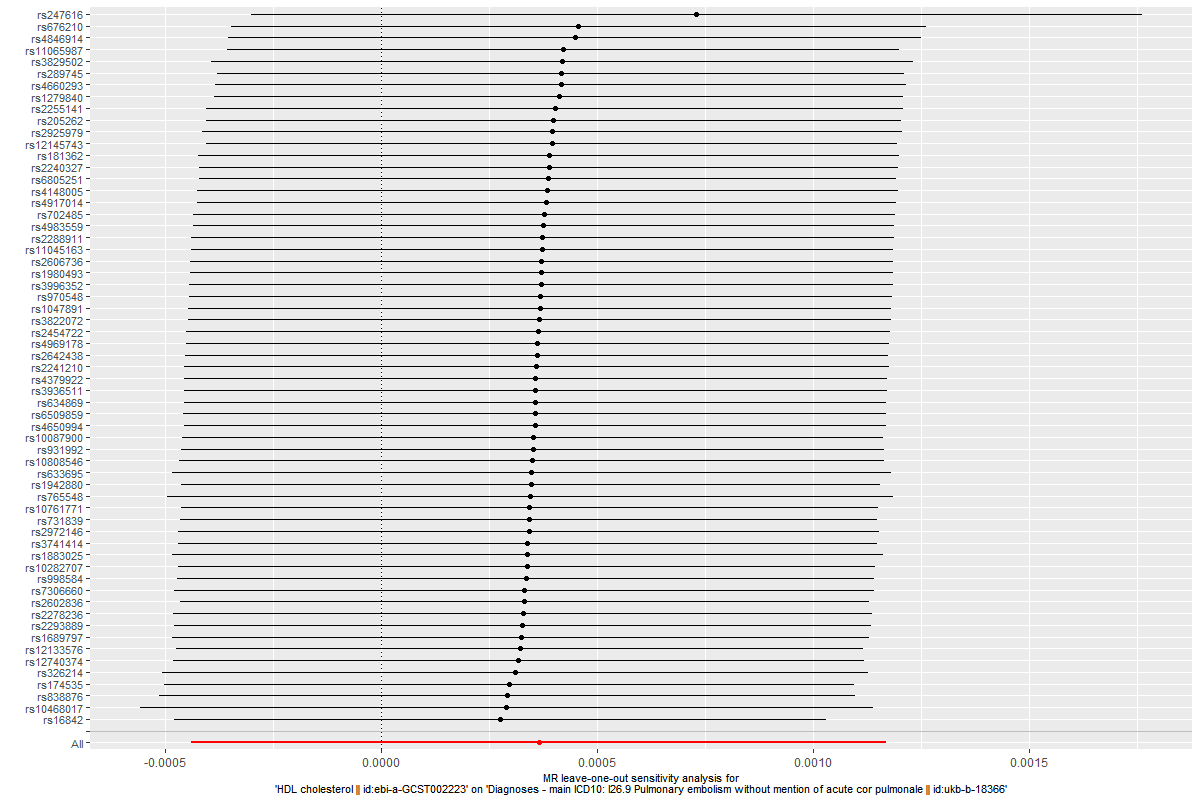


**Supplemental Figure S18: Leave-one-out test plot of the causal association between TG and PE**


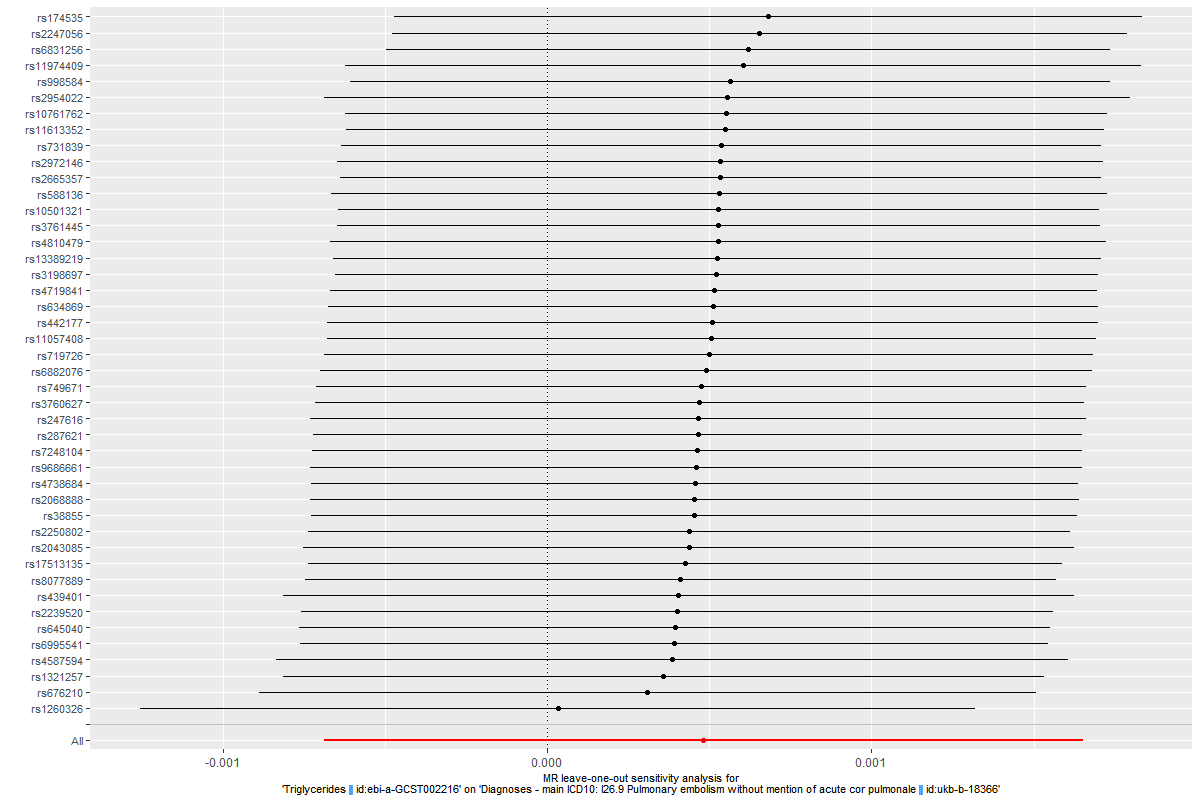


**Supplemental Figure S19: Scatterplot of the causal relationships between DVT and LDL**


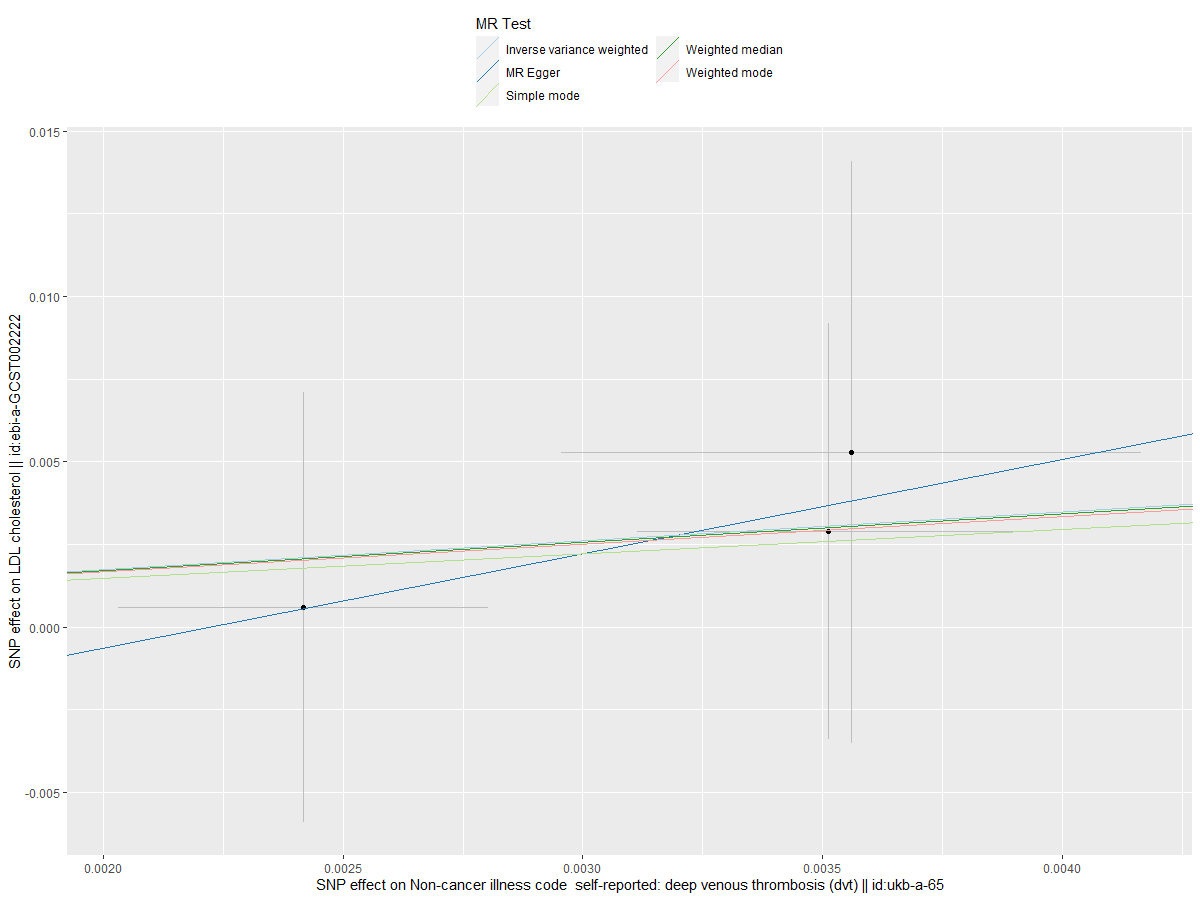


**Supplemental Figure S20: Scatterplot of the causal relationships between PE**

**and LDL**

**
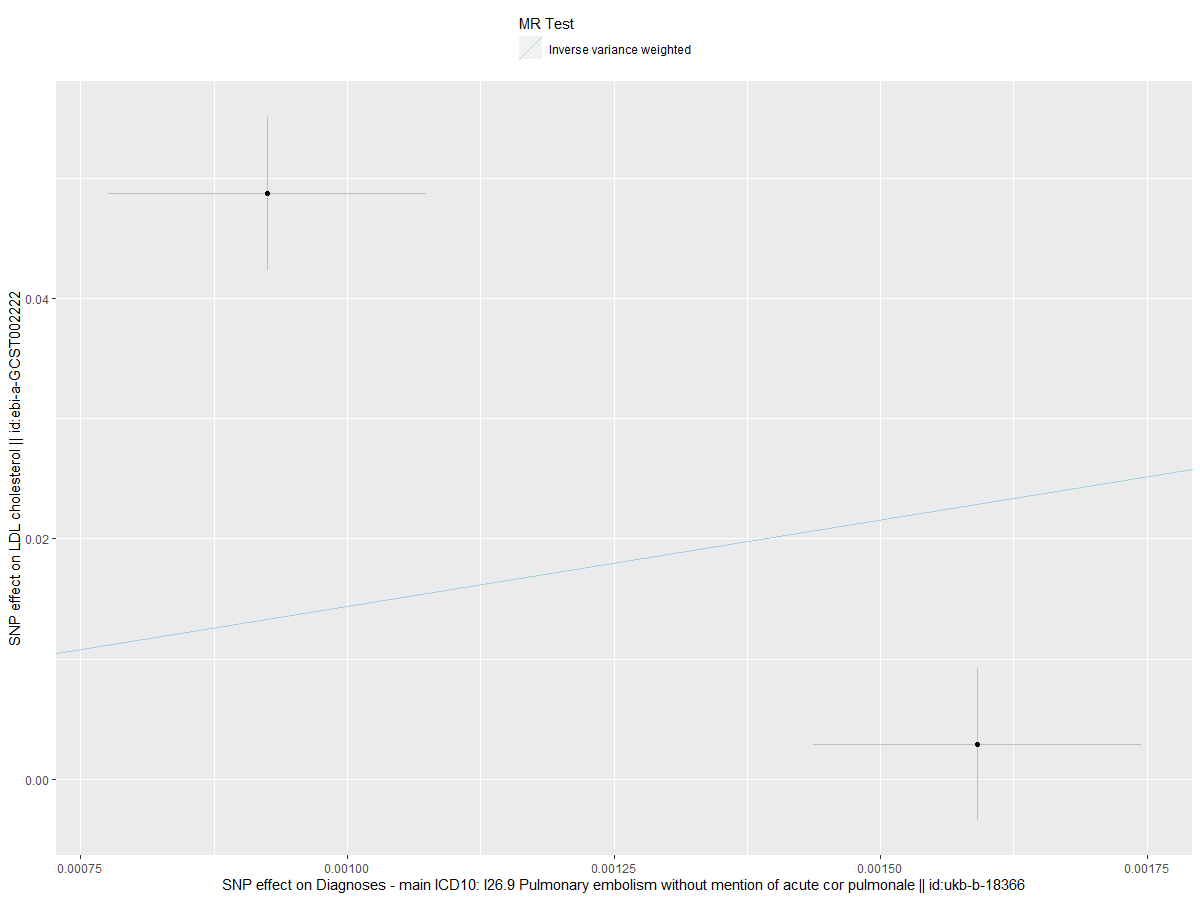
**

**Supplemental Figure S21: Scatterplot of the causal relationships between DVT and HDL**

**
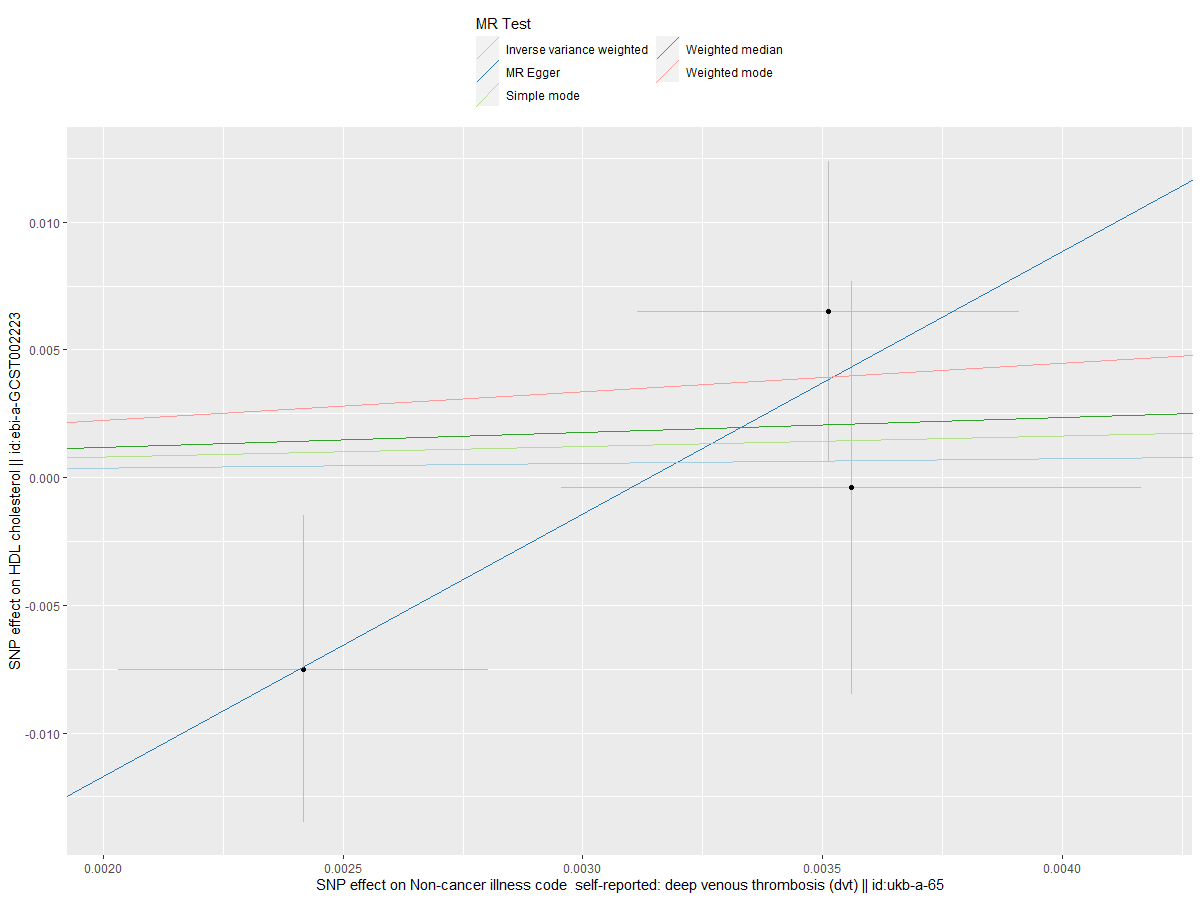
**

**Supplemental Figure S22: Scatterplot of the causal relationships between PE and HDL**

**
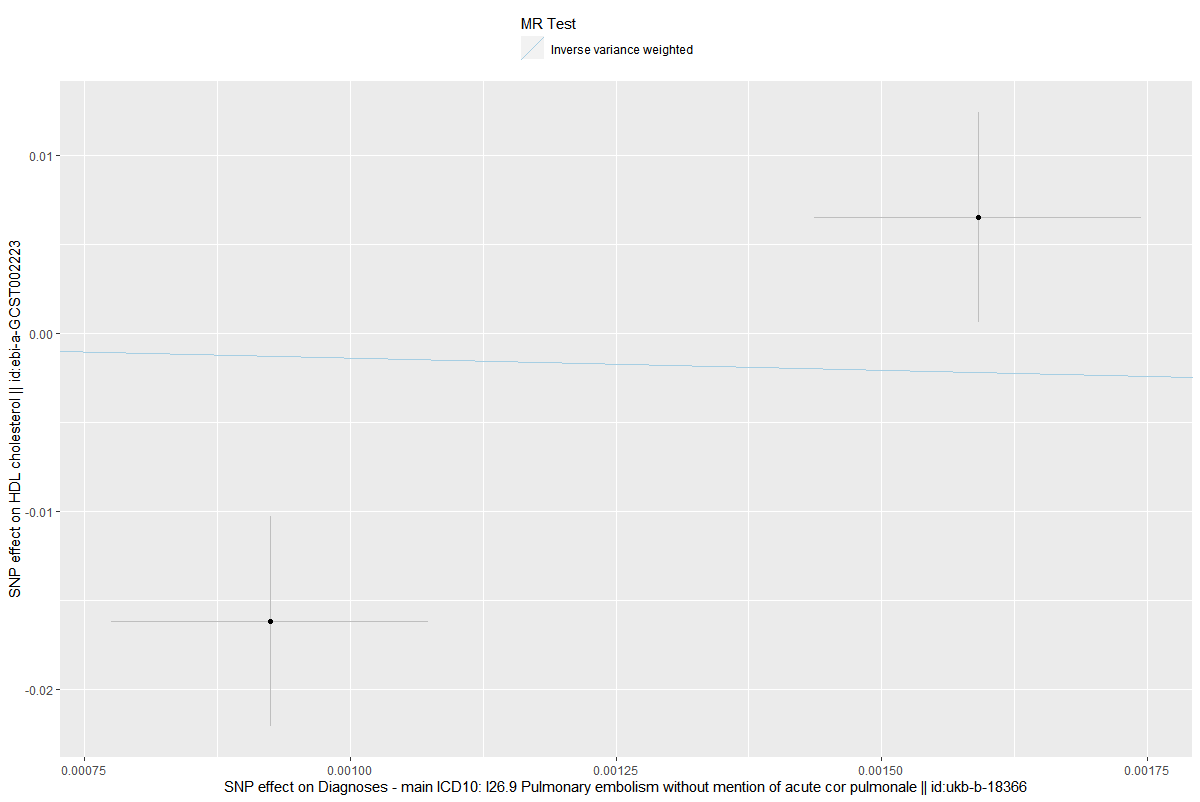
**

**Supplemental Figure S23: Scatterplot of the causal relationships between DVT and TG**

**
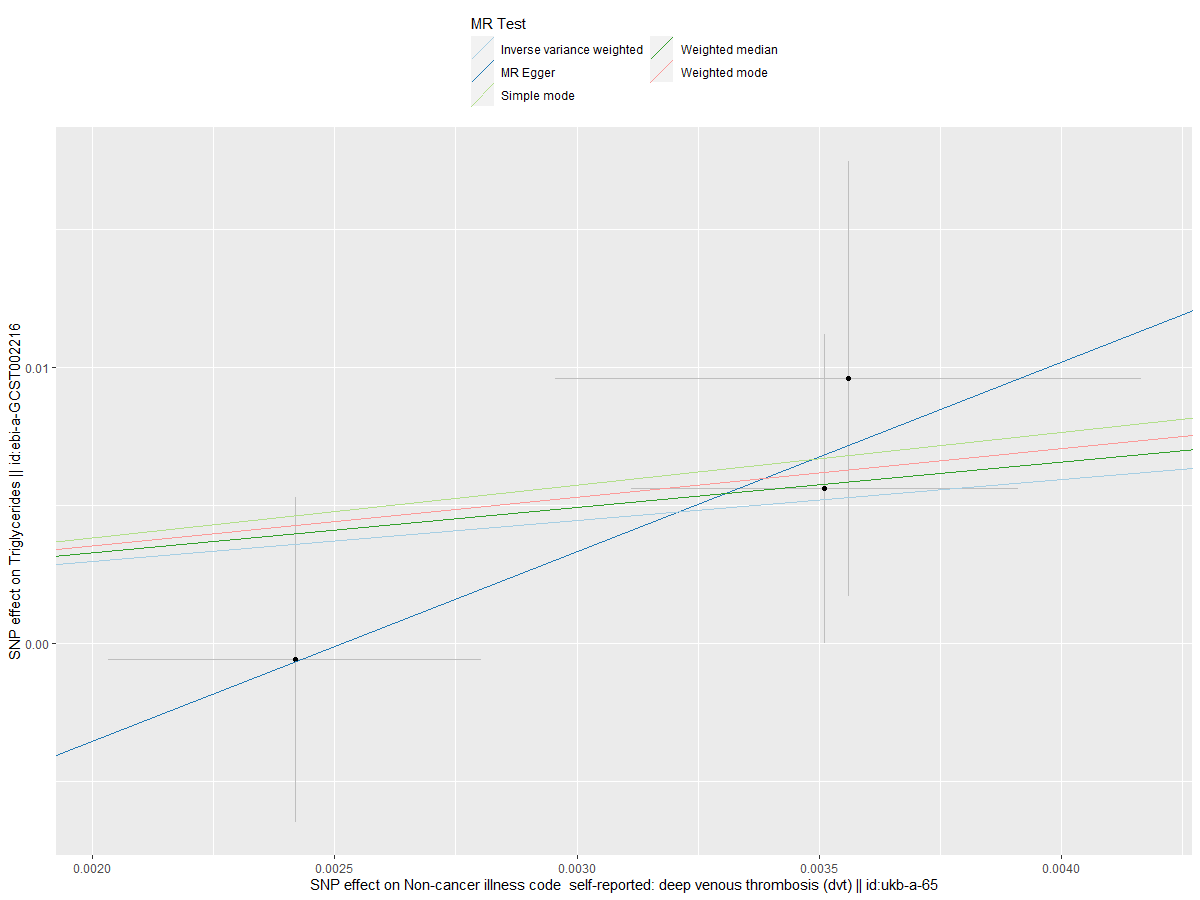
**

**Supplemental Figure S24: Scatterplot of the causal relationships between PE and TG**

**
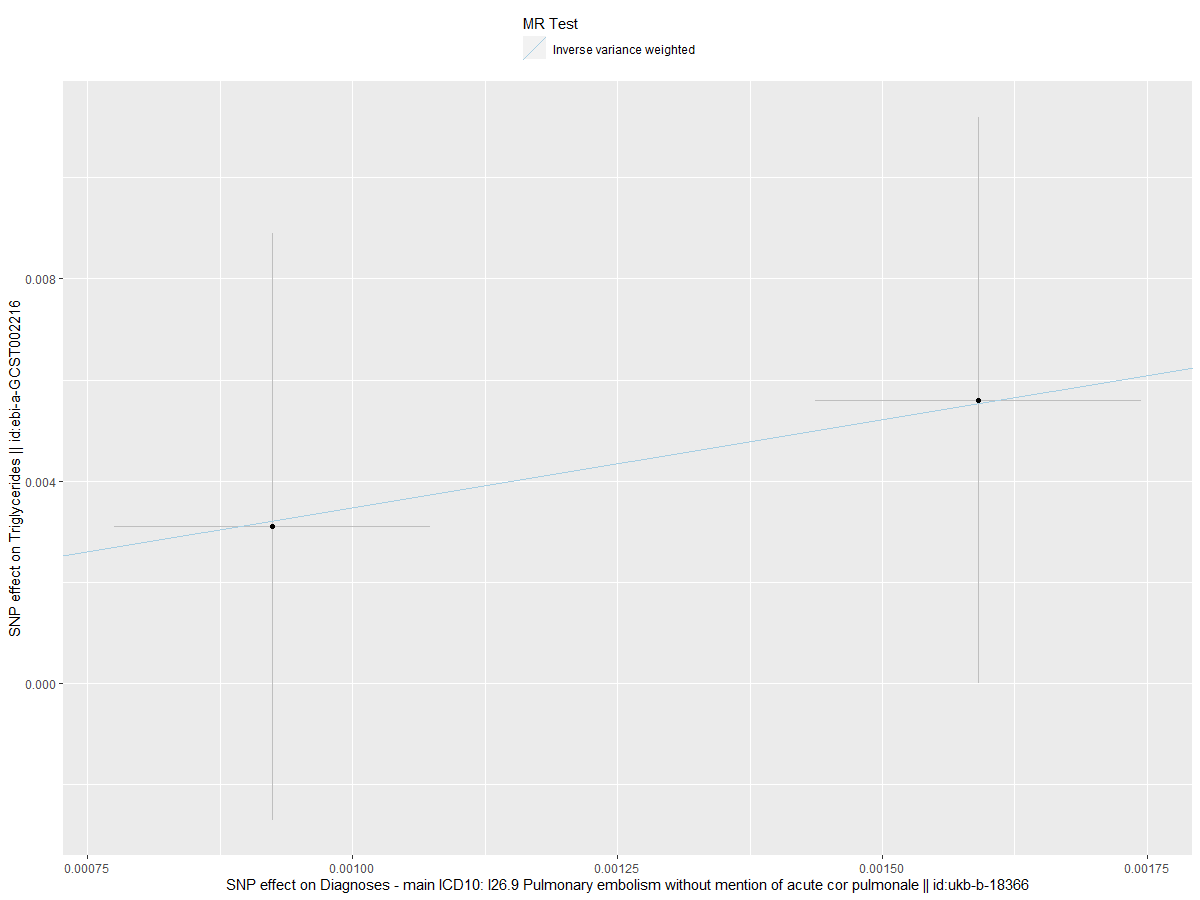
**

**Supplemental Figure S25: Leave-one-out test plot of the causal association between DVT and LDL**

**
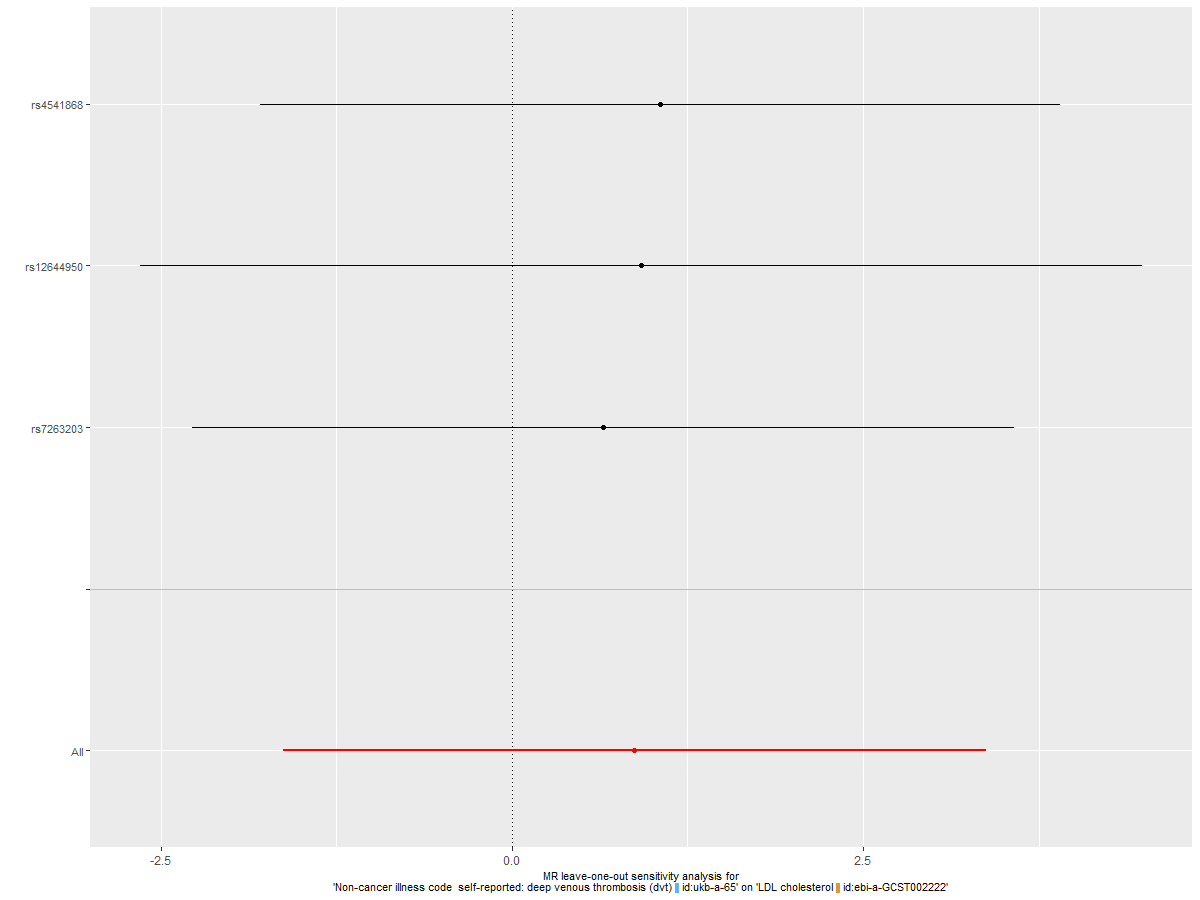
**

**Supplemental Figure S26: Leave-one-out test plot of the causal association between DVT and HDL**

**
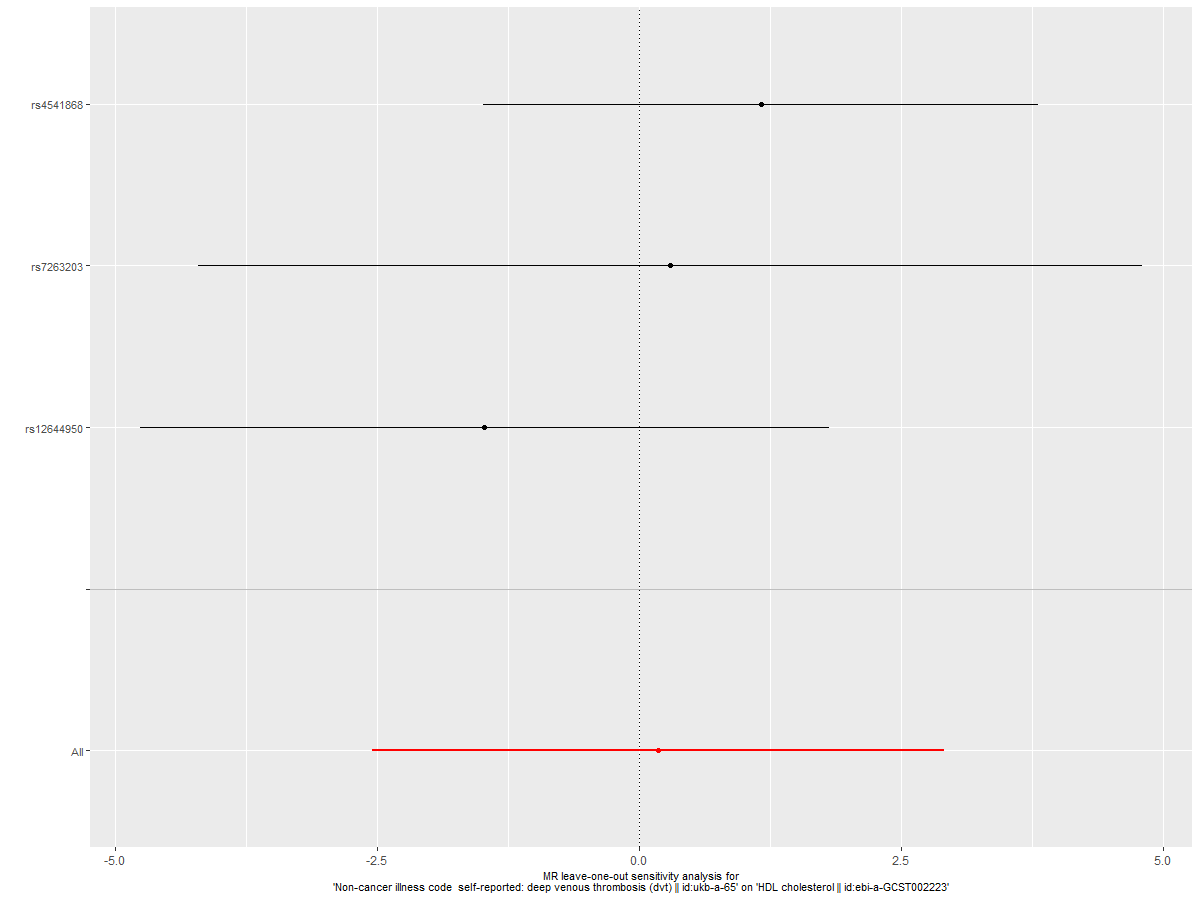
**

**Supplemental Figure S27: Leave-one-out test plot of the causal association between DVT and TG**

**
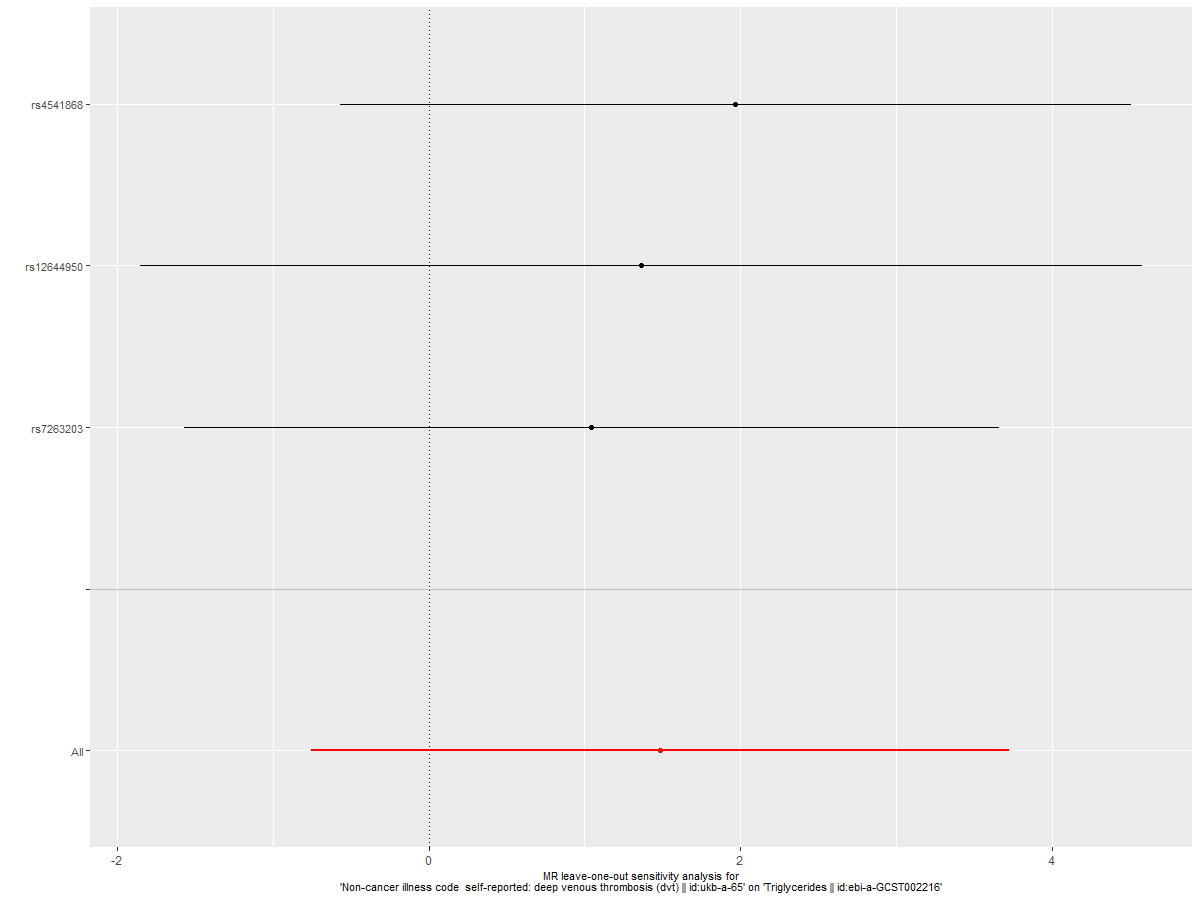
**
